# Factor-based deep reinforcement learning for asset allocation: Comparative analysis of static and dynamic beta reward designs

**DOI:** 10.1371/journal.pone.0332779

**Published:** 2025-12-30

**Authors:** Nak Hyun Jung, Taeyeon Oh

**Affiliations:** Seoul AI School, aSSIST University, Seoul, Republic of Korea; University of Almeria: Universidad de Almeria, SPAIN

## Abstract

Traditional asset allocation rules, while effective in stable phases, tend to erode once markets enter volatile regimes or undergo structural breaks. Research in deep reinforcement learning (DRL) has usually emphasized raw-return rewards, leaving aside the role of factor exposures (β) that shape both risk-adjusted payoffs and adaptive responses.

This paper advances a Factor-based Deep Reinforcement Learning for Asset Allocation (FDRL) framework in which β sensitivities—estimated via rolling regressions on momentum, volatility, deviation, and volume signals—inform both the state representation and the reward design. Five reward variants are examined (Sharpe, Sortino, Static-β, Dynamic-β, Momentum-β) using PPO, SAC, and TD3 across equities, cryptocurrencies, macroeconomic instruments, and mixed portfolios.

Empirically, β-based rewards generate heterogeneous but interpretable patterns. In equities, Dynamic-β improves annualized returns from roughly 20% (Sharpe baseline) to 23–24%, with Sharpe rising from 1.04 to about 1.27 across windows. In cryptocurrencies, Dynamic-/Momentum-β achieve 38–43% annual returns but remain highly regime-sensitive, with drawdowns often exceeding –35%. In macro instruments, Static-β delivers the most stable behaviour, maintaining volatilities near 8–9% and limiting drawdowns to roughly –18%. In mixed-asset portfolios, Momentum-β under TD3 produces the strongest gains (cumulative returns above 70–80%), exceeding equal-weight baselines whose CAGR remains near 19–22% with Sharpe ratios around 1.25.

All findings were validated through beta-window sensitivity checks (30/60/90/120 days), regime-conditional analysis, and multiple robustness tests including HAC, Wilcoxon, jackknife Sharpe, moving-block bootstrap, and false-discovery-rate adjustments. These diagnostics confirm that the main performance patterns are not driven by window choice or serial dependence.

Four contributions follow. First, a reward structure operationalizing time-varying β. Second, systematic benchmarking of factor-sensitive objectives. Third, evidence on asymmetric outcomes across asset classes. Finally, a framework that reconciles responsiveness with interpretability and risk discipline in allocation.

## Introduction

Deep Reinforcement Learning (DRL) has reshaped quantitative portfolio allocation by enabling policies that adapt to abrupt shifts in market regimes [1,2]. Earlier paradigms grounded in mean–variance optimization [3] and multifactor asset pricing [4] offered valuable structure, yet rested on static linear assumptions. Such assumptions have difficulty representing volatility clustering, asymmetric responses, and the nonlinear dependencies evident in modern markets, particularly in volatile segments such as digital assets [5].

Factor-based investing links theoretical asset pricing to observed return patterns by decomposing performance into systematic drivers including momentum, volatility, and size [6,7]. However, most DRL applications still optimize rewards around singular metrics such as the Sharpe or Sortino ratio, without explicit control over factor exposures [2,8]. Embedding factor sensitivities (β) directly into the reward function remains underexplored [9].

The framework developed here incorporates three forms of β-driven objectives. Static-β captures fixed regression-based sensitivity to a set of technical indicators. Dynamic-β allows for time-varying responsiveness, estimated via 30-day rolling regressions on momentum, volatility, moving average deviation, and volume Z-score. Momentum-β isolates exposure to momentum as a standalone reward driver, enabling a sharper view of factor-specific policy learning. All variants are integrated into the state vector and reward computation, alongside a Sharpe-based baseline.

Evaluation is performed within a Proximal Policy Optimization (PPO) setting on a diversified universe of equities, cryptocurrencies, macroeconomic instruments, and blended multi-asset portfolios [10–12]. This structure enables a direct comparison of reward formulations, each tied to economically interpretable signals, under varied market regimes.

This study frames its investigation around two primary questions. First, does embedding factor exposure into the reward structure provide tangible advantages over return-only objectives in terms of portfolio performance and policy stability? Second, among the three beta formulations—static, dynamic, and momentum-oriented—which configuration proves more responsive to structural shifts and regime transitions in diverse market environments?

The study contributes by constructing an RL reward design explicitly anchored in factor economics, producing cross-asset evidence on how β alignment affects allocation, and illustrating a pathway toward more transparent, regulator-ready AI allocation systems [13–15].

## Related works

### AI-driven asset allocation

Artificial intelligence has become central to portfolio design. Reinforcement learning (RL) and deep learning (DL) capture non-linearities, high dimensionality, and regime shifts [1]. Experiments with DDPG, SAC, and PPO suggest that model-free RL adapts well under non-stationarity, while average-reward policy iteration provides horizon-sensitive trade-offs [14]. Yet no universal winner emerges; adaptability depends on context.

Applications are diverse. Deep Q-Networks have been extended to handle microstructure and trading costs [16]. In crypto markets, state spaces enriched with technical and sentiment signals achieved excess returns above 20% [5]. Multi-agent RL (MARL) expands this scope: masked reconstruction for dimensionality reduction [17], game-theoretic MARL for cooperation and competition [18], and modular MARL scaling to high-cardinality portfolios [19]. Still unresolved is whether these designs generalize across asset classes.

Hybrid approaches connect DL forecasts with classical optimizers. Some integrate neural predictors into mean–variance or Omega ratio [20]; others link ML regressors with mean–VaR to address tail risk [21]. Interpretability remains a limitation [8]. Orthogonal reward decomposition has been proposed as a way to balance competing objectives and improve robustness [13].

Language models have also entered finance. BERT transforms news and sentiment into tradable features [22]. MARL has been tested for cross-asset risk, and Pareto-based allocation for multi-objective trade-offs [18]. Alongside, attention maps, SHAP, and LIME provide interpretability tools [23,24]. Still, transparency remains fragmented and case-specific.

A deeper challenge concerns alignment with financial theory. Comparative reviews note methodological heterogeneity [8,25], while heuristic evolutionary optimizers remain competitive [26]. Hansen [27] warns of a “paradox of uncertainty”: AI mitigates risks yet introduces new forms of model risk.

This study differs from prior work that merely inserted factor signals into state definitions. Here, reward functions are tied to time-varying factor exposures, treating β not as exogenous input but as the optimization target itself.

### Traditional asset allocation theories and factor-based models

Portfolio theory originates with Markowitz’s Modern Portfolio Theory (MPT), showing that diversification reduces idiosyncratic risk by balancing correlations [3]. Sharpe extended this with the CAPM, placing market beta (β) at the core of systematic risk [28]. Elegant though it was, the single-factor frame proved too restrictive.

Subsequent work broadened the scope. Fama–French added firm size (SMB) and value (HML) to capture beta’s weak explanatory power [4]. Carhart introduced momentum [29], while Campbell distinguished “bad” volatility-driven from “good” long-term beta [9]. These additions refined interpretation, yet the proliferation of factors underscored theoretical instability.

Alternative risk definitions deepened the debate. Sortino advocated downside deviation against a minimum return [30]. Harvey later warned of a “factor zoo,” noting that many claimed factors lack economic grounding [7]. Not all statistical regularities qualify as true risk sources.

Machine learning (ML) further reshaped the field. ML highlights high-dimensional non-linear patterns [8], but Ang [31] stressed that factor models often exaggerate explanatory power. Predictive accuracy alone—without economic rationale—can mislead [10].

Evidence is mixed. Deep networks embedded into factor models show gains [10], while reinforcement learning (RL) reframes allocation as sequential choice. Cornalba (2024) linked multi-objective rewards with portfolio constraints [13], and hybrid RL–quantum methods explored adaptive weighting [32]. Robustness, however, remains contested.

Explainability is a parallel concern. Finance-specific taxonomies for XAI [24] and policy visualization in RL [33] show partial progress. Still unresolved is a core point: very few RL models treat β sensitivity itself as a learning target. In shifting regimes, this gap is far from trivial.

### Beta estimation and reward design based on technical factors

Ross’s Arbitrage Pricing Theory (APT) framed returns as linear factor combinations [34]. In contrast, this study emphasizes technical indicators as dynamic signals for DRL. Momentum, long central in Carhart’s four-factor model [29], retains relevance under regime shifts [35], making it a natural input for time-varying beta.

Beta (β) reflects market sensitivity, but fixed-window regressions often diverge—by nearly 40% in high-frequency cases [11]. Dynamic methods address such breaks [12]. Campbell’s notion of “good” and “bad” beta [9] extends here: the former tied to stable exposures, the latter to volatility shocks.

In DRL portfolios, β can enter the state vector. Prior studies used Q-learning with moving averages or volume [16], and beta-neutral designs outperformed static OLS by nearly 30% [2]. Other works linked forecasts with mean–VaR [21], or broadened scope through sentiment and graph features [15,22,36]. Multi-agent settings often embed many indicators [18,37].

Reward construction follows similar logic. Some embed β directly into returns [1,25]; others penalize drawdown or concentration [19]. Multi-objective rewards improved efficiency, in some reports tripling baseline results [13]. In volatile domains like crypto, Sharpe–drawdown blends guided allocation [5].

Challenges remain. Overfitting and interpretability persist [24]. Still, linking rolling beta estimates with structured rewards bridges factor theory and adaptive RL. Unlike neutral or purely predictive rules, the proposed design embeds β in both state and reward, spanning multiple assets and algorithms.

### Summary of related works and research gap

RL-based portfolio research has advanced along divergent paths rather than a single trajectory. Park and Sim (2020) combined technical and sentiment inputs, while Lin and Chen (2022) designed multi-agent systems for faster learning. By contrast, Behera (2023) and Ma (2021) imposed explicit risk limits, and Hambly (2023) with Cornalba (2024) highlighted multi-objective rewards, showing that stability under frictions can rival pure return targets. The field lacks consensus.

Factor investing—anchored in size, value, and momentum since Fama (1996)—intersects unevenly with these approaches. Deep models capture shifting exposures (Feng, 2024), but Bagnara (2024) warns that many ML studies privilege fit over economic meaning. Interpretation diverges, and what qualifies as a “factor” remains unsettled.

Explainability further complicates the picture. Graph tools and SHAP improve transparency at prediction level [36], yet Vouros et al. (2022) argue that sequential policy paths remain opaque [33]. Output clarity does not resolve decision ambiguity. Evidence is fragmented.

Two research gaps follow. First, RL rarely embeds classical factors—such as Fama–French variables—into state or reward functions. Second, dynamic factor models seldom connect with RL structures. Both limit theoretical grounding.

This study addresses these issues by integrating rolling-regression betas into factor-aware rewards, while state vectors blend technical and factor signals. The design links three strands: RL allocation (Hambly, 2023), factor modeling (Fama, 1996), and interpretability (Weber, 2024). It balances adaptability with interpretability, aiming to test sensitivity under regime shifts.

Still unresolved is how to encode economic structure without undermining operational performance (Bagnara, 2024). Future work may examine hierarchical factors, new asset classes, and policy-level XAI.

## Methodology

### Model design and theoretical framework

Factor integration reshapes a conventional reinforcement learning framework. The cycle of observation (*S*_*t*_), action (*A*_*t*_), and reward (*R*_*t*_) remains, but its meaning is reinterpreted. Here, factors are not exogenous statistics—they are embedded within both state representation and reward design. Three reward alternatives are explored. A Static-β version fixes regression sensitivities over time. A Dynamic-β form adjusts exposures through rolling regressions of momentum, volatility, moving-average deviations, and volume signals. Finally, Momentum-β focuses only on momentum, acting as a minimal benchmark. Each reflects a different trade-off: the first stresses stability, the second adaptability, and the third parsimony.

Rewards do more than register returns. The environment delivers realized gains, but the agent evaluates outcomes through Sharpe, Sortino, or β-linked measures. This separation is deliberate. It prevents confusion between what markets generate and how performance is assessed. A small but crucial distinction.

Portfolio allocation is constrained: weights are non-negative and normalized via a softmax transformation. Training relies on Proximal Policy Optimization (PPO). The clipped objective stabilizes learning while allowing smoother updates than many off-policy methods. The choice is pragmatic, not merely conventional.

**Fig 1:**
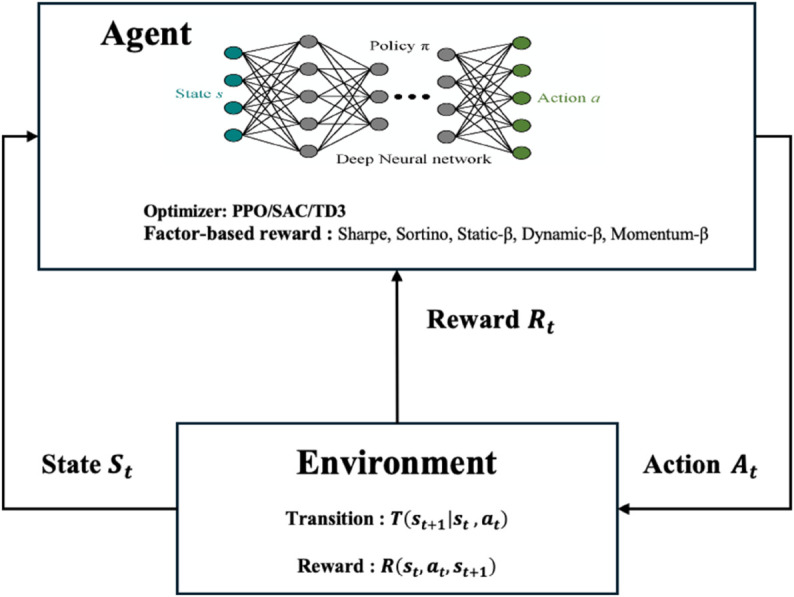
Factor-Based Deep Reinforcement Learning (FDRL) framework.

As illustrated in Fig 1, factor signals feed into both state and reward. The agent makes allocations, the environment responds, and the loop continues. The form looks familiar, yet its essence is different: learning itself is guided by embedded factors. This is not trivial.

### Factor definition and β estimation

In this study, we define time-varying factor betas $\beta_{i,t}$ as the sensitivity of asset *i*’s return to a set of standardized technical indicators. Unlike traditional models such as CAPM [28] or Fama-French [6], which assume static market betas, we estimate a Dynamic-β vector using 30-day rolling regressions. This approach is based on the theoretical structure of the Arbitrage Pricing Theory (APT) proposed by Ross (1976) [34], which models asset returns as a linear combination of multiple factor sensitivities and their corresponding risk premia. We extend this framework by treating technical indicators as time-varying factors and proposing a method to dynamically estimate their sensitivities.

The vector of factor sensitivities for asset *i* at time *t* is defined in Eq (1):

$$\beta_{i,t}={\left[\beta_{i,1,t},\;\beta_{i,2,t},\;\ldots ,\;\beta_{i,K,t}\right]}^{⊤}\in {\mathbb{R}}^{K}$$ (1)

Each coefficient $\beta_{i,k,t}$ is estimated via the linear regression model in Eq (2):

$$r_{i,t}=\alpha_{i}+\sum_{k=1}^{K}\beta_{i,k,t}\cdot f_{i,k,t}+\varepsilon_{i,t}$$ (2)

Here, *r*_*i*,*t*_ is the log return of asset *i* at time *t*, *f*_*i*,*k*,*t*_ is the standardized value of the *k*-th technical factor, and $\beta_{i,k,t}$ is the estimated sensitivity (beta) to that factor. The factor vector consists of *K* = 8 elements. Below, we define each technical factor *f*_*i*,*k*,*t*_, along with its economic interpretation:

**(1) Momentum Factor** $f_{i,t}^{\text{mon}}$: Momentum is defined as the 20-day price return, as shown in Eq (3):
  $$f_{i,t}^{\text{mon}}=\frac{P_{i,t}}{P_{i,t-20}}-1$$ (3)
  where *P*_*i*,*t*_ is the closing price of asset *i* at time *t*. This factor measures trend-following behavior.
**(2) Volatility Factor** $f_{i,t}^{\text{vol}}$: Volatility is measured as the standard deviation of returns over the 20-day window, as shown in Eq (4):
  $$f_{i,t}^{\text{vol}}=\text{std}(r_{i,t-20:t})$$ (4)
  This reflects the traditional measure of market risk.
**(3) Moving Average Deviation** $f_{i,t}^{\text{ma\_dev}}$: The deviation from the 20-day simple moving average (SMA) is defined in Eq (5):
  $$f_{i,t}^{\text{ma\_dev}}=\frac{P_{i,t}-MA_{i,t}^{\left(20\right)}}{MA_{i,t}^{\left(20\right)}}$$ (5)
  This indicator is useful for detecting mean-reversion tendencies and identifying overbought or oversold conditions.
**(4) Volume Z-score** $f_{i,t}^{\text{vol\_z}}$: As illustrated in Eq (6), the volume Z-score normalizes current volume by its historical mean and standard deviation:
  $$f_{i,t}^{\text{vol\_z}}=\frac{V_{i,t}-{\bar{V}}_{i,t}}{s_{V,i,t}}$$ (6)
  This factor captures volume anomalies and the strength of market reaction.
**(5) Sharpe Ratio** $f_{i,t}^{\text{sharpe}}$: The Sharpe ratio, shown in Eq (7), quantifies risk-adjusted return:
  $$f_{i,t}^{\text{sharpe}}=\frac{\mu_{i,t}^{\left(20\right)}}{\sigma_{i,t}^{\left(20\right)}+\epsilon }$$ (7)
  where μ is the mean return, σ is the standard deviation, and ϵ is a small constant for numerical stability.
**(6) Maximum Drawdown (MDD)** $f_{i,t}^{\text{mdd}}$: Based on Eqs (8, 9), the drawdown factor is normalized in Eq (10):
  $$C_{i,s}=\prod_{k=t-20}^{s}(1+r_{i,k})$$ (8)
  $${\text{MDD}}_{i,t}=\min_{s\in [t-20,t]}\left(\frac{C_{i,s}-\max_{u\in [t-20,s]}C_{i,u}}{\max_{u\in [t-20,s]}C_{i,u}}\right)$$ (9)
  Normalized to:
  $$f_{i,t}^{\text{mdd}}=1-|{\text{MDD}}_{i,t}|$$ (10)
  This factor penalizes large losses, thus encouraging risk-averse strategies.
**(7) Equal-Weighted Market Beta** $f_{i,t}^{\text{eqw\_price}}$: This factor represents the sensitivity of asset *i*’s return to an equal-weighted market index return. It is estimated through a 30-day rolling OLS regression as shown in Eq (11):
  $$f_{i,t}^{\text{eqw\_price}}={\hat{\beta }}_{i,t}^{\text{eqw}},\;r_{i,t}=\alpha_{i}+\beta_{i,t}^{\text{eqw}}\cdot r_{t}^{\text{eqw}}+\varepsilon_{i,t}$$ (11)
  where $r_{t}^{\text{eqw}}$ is the return of the equal-weighted market index.
**(8) Policy Portfolio Beta** $f_{i,t}^{\text{eqw\_policy}}$: This factor reflects the asset’s exposure to the return of the agent’s previously executed portfolio. It is defined by the regression in Eq (12):
  $$f_{i,t}^{\text{eqw\_policy}}={\hat{\beta }}_{i,t}^{\text{policy}},\;r_{i,t}=\alpha_{i}+\beta_{i,t}^{\text{policy}}\cdot r_{t}^{\text{policy}}+\varepsilon_{i,t}$$ (12)
  where $r_{t}^{\text{policy}}$ is the portfolio return generated by the agent’s past policies up to time *t*.

Each estimated coefficient $\beta_{i,k,t}$ is embedded in the RL agent’s state vector. For each asset *i* at time *t*, the complete state vector is constructed as shown in Eq (13):

$${𝐬}_{i,t}=\left[f_{i,t}^{\text{mon}},\;f_{i,t}^{\text{vol}},\;f_{i,t}^{\text{ma\_dev}},\;f_{i,t}^{\text{vol\_z}},\;f_{i,t}^{\text{sharpe}},\;f_{i,t}^{\text{mdd}},\;f_{i,t}^{\text{eqw\_price}},\;f_{i,t}^{\text{eqw\_policy}}\right]$$ (13)

This 8-dimensional vector captures trend, risk, liquidity, and exposure-based signals, enabling interpretable and FDRL. For a portfolio of *N* assets, the full input to the RL agent forms an N×8 matrix, facilitating cross-asset comparison and consistent learning across time.

The policy factor was defined as the cumulative return generated by the agent’s own portfolio up to time *t*–1. No information beyond *t*–1 is incorporated when computing this measure, which prevents look-ahead bias. At each decision point the order of operations is fixed: market observation, policy action, trade execution, and then the update of the state representation. All subsequent estimates are therefore conditional only on past and present information, never on future outcomes.

Eight technical factors are employed to characterize asset behavior, each computed on a rolling 20-day basis: momentum, volatility, moving-average deviation, volume Z-score, Sharpe ratio, maximum drawdown, equal-weighted return, and equal-weighted policy return. All metrics are standardized using Z-scores to maintain scale comparability.

Factor sensitivities (β) are estimated via 30-day rolling OLS regression (Eq 14):

$$r_{i,t}=\alpha_{i}+\sum_{k=1}^{K}\beta_{i,k,t}\cdot f_{k,t}+\varepsilon_{i,t}$$ (14)

where *r*_*i*,*t*_ denotes the log return of asset *i* at time *t*, *f*_*k*,*t*_ is the *k*-th factor value, and $\beta_{i,k,t}$ is its estimated exposure. The regression employs *K* = 8 core technical indicators as predictors.

### Reward function design

The reinforcement learning agent selects portfolio weights ${𝐰}_{t}\in {\mathbb{R}}^{N}$ at each decision point. Here, *N* is the number of tradable assets. The portfolio-level exposure to systematic factors at time *t*, denoted $\beta_{\text{portfolio},t}$, is obtained as the weighted aggregation of asset-level betas (Eq 15):

$$\beta_{\text{portfolio},t}=\sum_{i=1}^{N}w_{i,t}\cdot \beta_{i,t}.$$ (15)

The design of the reward function is central to shaping the agent’s behavior. Five alternatives were examined, representing distinct views of portfolio efficiency and factor exposure.

#### (1) Sharpe-based Reward.

Defined in Eq 16, this formulation evaluates expected excess return relative to its total volatility:

$$r_{t}^{\text{Sharpe}}=\frac{𝔼[R_{t}-R_{f}]}{\sigma (R_{t}-R_{f})}.$$ (16)

*R*_*t*_ is the realized portfolio return, while *R*_*f*_ denotes the risk-free rate, assumed zero in our baseline. This ratio (Eq 16) has long served as a reference point for risk-adjusted optimization.

#### (2) Sortino-based Reward.

The Sortino ratio isolates downside risk, replacing the variance term with downside deviation (Eq 17):

$$r_{t}^{\text{Sortino}}=\frac{𝔼[R_{t}-R_{f}]}{\sigma_{\text{down}}(R_{t}-R_{f})}.$$ (17)

The denominator is defined in Eq 18:

$$\sigma_{\text{down}}(R_{t}-R_{f})=\sqrt{\frac{1}{T}\sum_{t=1}^{T}\min(R_{t}-R_{f},0{)}^{2}}.$$ (18)

Only negative returns relative to the threshold contribute. By penalizing shortfall events while ignoring gains, this measure (Eq 18) addresses the asymmetry in investor preferences.

Sharpe and Sortino-based rewards were estimated on rolling windows of past returns (20 days in baseline, 60 and 252 days for robustness). Returns were measured as log differences. Volatility and downside volatility were computed with equally weighted sample estimators, updated online.

#### (3) Dynamic-βbeta Reward.

Factor exposures fluctuate with market conditions. To capture this, dynamic betas were estimated from rolling regressions. The reward, given in Eq 19, emphasizes portfolios whose factor exposure exceeds that of a naïve equal-weight allocation:

$$r_{t}^{\text{dynamic-}\beta }=\max\left(\sum_{i=1}^{N}w_{i,t}\;\beta_{i,t},\;\sum_{i=1}^{N}w_{i}^{\text{eqw}}\;\beta_{i,t}\right),$$ (19)

where $w_{i}^{\text{eqw}}=1/N$. This specification (Eq 19) rewards strategies that adaptively exploit evolving factor structures.

#### (4) Static-βbeta Reward.

Some portfolio objectives require stability. The static reward aligns the portfolio with predefined factor targets as in Eq 20:

$$r_{t}^{\text{static-}\beta }=\sum_{k=1}^{K}\lambda_{k}(\sum_{i=1}^{N}w_{i,t}\;\beta_{i}^{\left(k\right)}).$$ (20)

Here, $\beta_{i}^{\left(k\right)}$ is the exposure of asset *i* to factor *k*, and $\lambda_{k}$ encodes the desired orientation. Eq 20 encourages controlled alignment with theoretical or policy-driven benchmarks.

#### (5) Momentum-βbeta Reward.

Factor exposures are not only levels but trajectories. Momentum in factor sensitivity is captured through incremental changes (Eq 21):

$$r_{t}^{\text{momentum-}\beta }=\sum_{i=1}^{N}w_{i,t}\;(\beta_{i,t}-\beta_{i,t-1}).$$ (21)

Positive contributions occur when portfolio weights increase exposure to factors showing rising influence. This specification (Eq 21) highlights directional adjustments.

Taken together, the five reward functions form a spectrum. Sharpe and Sortino emphasize efficiency of return relative to risk (Eqs 16–18). Static- and Dynamic- β rules (Eqs 19–20) represent anchored versus adaptive alignment. Momentum-β (Eq 21) highlights directional change. Their joint evaluation provides a structured comparison of portfolio objectives under reinforcement learning.

To facilitate interpretability and contrast the five reward formulations, we summarize their structural properties in Table 1. Each formulation exhibits a distinct orientation with respect to performance emphasis, volatility sensitivity, and regime adaptation. This overview clarifies the alignment between reward configuration and agent behavior under varying market conditions.

**Table 1:** Comparison of reward structures.

Reward Type	Core Objective	Risk Sensitivity	Notable Characteristics
Sharpe-based	Maximize excess return per unit of total volatility	Symmetric to both gain and loss	Simple, widely adopted, ignores downside asymmetry and factor exposure
Sortino-based	Focus on downside-adjusted return	Penalizes only negative deviation	Reflects investor risk aversion; stable in directional markets but unresponsive to structural change
Static-β	Align portfolio with fixed factor loadings	Dependent on initial λ vector	Enhances interpretability; may underperform under rapid regime shifts
Dynamic-β	React to evolving factor structure using rolling regression	High temporal sensitivity	Tracks market changes; susceptible to noise in short-term beta estimates
Momentum-β	Reward directional change in β values	Conditional on β drift	Favors trend-following; amplifies recent factor shifts, may cause overshooting

### MDP construction

Portfolio allocation was cast as a Markov Decision Process $\left(S_{t},A_{t},R_{t},T,\pi \right)$ within a custom OpenAI Gym setup, enforcing full investment and transaction costs.

The state *S*_*t*_ collects standardized market signals—20-day momentum, volatility, moving-average gap, volume Z-score, Sharpe ratio, and drawdown—plus two factor exposures. One is equal-weighted, the other policy-driven, both estimated by 30-day rolling OLS.

Actions *A*_*t*_ are continuous portfolio weights ${𝐰}_{t}\in [0,1{]}^{N}$, normalized through a softmax so that $\sum_{i}w_{i,t}=1$.

Rewards follow five options: Sharpe, Sortino, momentum-β, static-β, and dynamic-β. Each emphasizes a different trade-off between growth, downside control, and factor sensitivity.

Transitions update prices and factors daily. The policy $\pi (a_{t}|s_{t})$, parameterized by a neural network, was trained with PPO using clipped objectives and entropy regularization. This offered stability while still adapting under frequent rebalancing.

### Policy network and optimization algorithm

The policy network is implemented as a multi-layer perceptron (MLP) that maps the N×8 state matrix into a portfolio weight vector ${𝐰}_{t}\in {\mathbb{R}}^{N}$. A softmax layer enforces the budget constraint, ensuring non-negative weights summing to one:

$${𝐰}_{t}=\text{softmax}({𝐳}_{t}),\;{𝐳}_{t}=f_{\theta }(S_{t}).$$ (22)

Here, $f_{\theta }$ denotes the parameterized policy mapping states into raw scores.

Training employs Proximal Policy Optimization (PPO) for stability and efficiency. Its clipped objective is

$${ℒ}^{\text{CLIP}}(\theta )={𝔼}_{t}[\min(r_{t}(\theta ){\hat{A}}_{t},\;\text{clip}(r_{t}(\theta ),1-\epsilon ,1+\epsilon ){\hat{A}}_{t})],$$ (23)

where $r_{t}(\theta )=\frac{\pi_{\theta }(a_{t}|s_{t})}{\pi_{\theta_{\text{old}}}(a_{t}|s_{t})}$ is the likelihood ratio and ${\hat{A}}_{t}$ the estimated advantage.

An entropy term augments the loss to prevent premature convergence:

$${ℒ}^{\text{total}}={ℒ}^{\text{CLIP}}-\beta \;{𝔼}_{t}[ℋ(\pi_{\theta }(\cdot |s_{t}))].$$ (24)

Network architecture, optimization hyperparameters, and data inputs remain fixed across experiments. The only variation is the reward specification, isolating the effect of reward shaping on policy learning and portfolio outcomes.

### Ethics statement

This study relies exclusively on publicly available, aggregated financial time-series data (daily prices and trading volumes) and does not involve human participants, individual-level records, or any sensitive personal information. In accordance with institutional and journal guidelines, no institutional review board (IRB) approval or informed consent was required.

## Experiments

This section outlines the series of tests conducted to demonstrate the effectiveness of our interpretable deep reinforcement learning framework. To ensure reproducibility and clarity, we provide complete access to both the dataset and implementation code (refer to Supporting information section for details). The dataset is provided as supplementary material (S1 Data), the code and data of the paper are available at Zenodo: https://doi.org/10.5281/zenodo.17639570.

### Dataset description

To empirically evaluate the proposed DRL-based asset allocation framework, we constructed a multi-layered dataset grounded in rigorous financial theory and practical applicability. As summarized in Table 2, the dataset consists of three main asset classes—equity, cryptocurrency, and macroeconomic assets—with an additional multi-asset configuration for robustness testing under heterogeneous conditions.

**Table 2:** Summary of asset classes and dataset configuration.

Asset	#	Constituents	Train Period	Test Period
Equity	22	AAPL, MSFT, GOOGL, AMZN, META, TSLA, NVDA, NFLX, JPM, V, DIS, INTC, BA, XOM, JNJ, KO, PG, WMT, UNH, SPY, QQQ, DIA	2015-01-02 ~ 2022-04-29 (70%)	2022-05-02 ~ 2025-06-27 (30%)
Crypto	4	BTC-USD, ETH-USD, BNB-USD, SOL-USD	2020-05-26 ~ 2023-12-13 (70%)	2023-12-14 ~ 2025-06-27 (30%)
Macro	5	TLT, IEF, GLD, SLV, TIP	2015-01-02 ~ 2022-04-29 (70%)	2022-05-02 ~ 2025-06-27 (30%)
Multi	28	Equity (22) + Macro (5) + BTC-USD (1)	2015-01-02 ~ 2022-04-29 (70%)	2022-05-02 ~ 2025-06-27 (30%)

The equity set covers 22 large-cap U.S. stocks, selected by market capitalization and liquidity. It combines the FANG+ group with major S&P 500 and Dow Jones constituents to secure sectoral diversity. Technology exposure includes Apple (AAPL), Microsoft (MSFT), and NVIDIA (NVDA); non-tech firms include JPMorgan Chase (JPM), ExxonMobil (XOM), and Coca-Cola (KO). Selection follows a multi-factor framework capturing size and value [4], with allocation balance informed by ML-based modeling [21]. The crypto set consists of Bitcoin (BTC-USD), Ethereum (ETH-USD), Binance Coin (BNB-USD), and Solana (SOL-USD). To avoid unstable early data, only post-June 2020 observations were retained [1]. Selection applied a top-market-cap filter [18] and correlation-based screening [36]. Macro coverage includes ETFs tied to interest rates, inflation, and commodities: TLT, IEF (U.S. Treasuries), GLD (gold), SLV (silver), and TIP (TIPS). They emphasize inflation-hedging roles [15]. Preprocessing used Dynamic-β estimation [11] and news-response models [12]. A combined 28-asset universe was formed by merging 22 equities, 5 macro ETFs, and BTC-USD. This construction tests cross-market adaptability and diversification of DRL strategies [2].

Daily prices from 2015-01-02 to 2025-06-30 were obtained via Yahoo Finance. Dates were aligned through common indexing, with missing values forward-filled [16]. Data reliability was checked against ML-based forecasting quality protocols [38].

All price and volume series were obtained from Yahoo Finance, using only publicly available, non-identifiable market data. The collection and analysis of these data complied with the terms and conditions of Yahoo Finance and did not involve any intervention or interaction with human participants.

### Preprocessing and feature engineering

Data could not be used in raw form. Prices and trading volumes from different assets were first placed on a single calendar to maintain consistency, and gaps were filled forward. This step, though minor, was essential to prevent artificial jumps. Indicators were then standardized, but with two distinct horizons: technical measures employed 20-day rolling z-scores to capture short swings, while β values relied on 30-day windows to reveal more persistent exposures. What emerged was the state vector. Rather than a simple stack of variables, it condensed recent market behavior and factor sensitivities into a form that the policy network could interpret for decision-making.

### Experimental setup

Yahoo Finance provided daily prices and volumes from Jan 2015 to Jun 2025. Series were aligned to trading days, with gaps forward filled. A small step, yet crucial. Data were split into training (2015–2022) and testing (2022–2025). Learning hinged on PPO. SAC and TD3 were only for comparison. Agents shared one architecture, implemented in Stable-Baselines3, trained for 50,000 steps with seed 42. Features came from two horizons: technical indicators standardized over 20 days, and Dynamic-β values from 30-day rolling regressions. These formed an N×8 state matrix—not just a pile of signals, but a compressed picture of market conditions. Trading costs were set at 10 bps per dollar traded; returns are reported net of costs. Turnover, $\frac{1}{2}\sum_{i}|w_{i,t}^{target}\;-\;w_{i,t-1}^{post}|$, was measured daily and annually. Returns are reported net of frictions.

### Baseline models and comparison strategies

The FDRL framework was evaluated against both traditional and learning-based references. As passive control, the Equal-Weighted Portfolio distributes identical shares across assets. The Mean–Variance Portfolio follows the Markowitz paradigm, relying on historical means and covariances to generate static allocations.

Within DRL, five variants were constructed according to reward design: Sharpe- and Sortino-based objectives, and three factor-oriented forms (Dynamic-β, Static-β, Momentum-β). Each variant emphasized a different trade-off, ranging from general risk-adjusted efficiency to explicit sensitivity to market factors. All experiments adopted uniform settings: daily rebalancing, a 0.1% transaction fee, and identical hyperparameters.

Performance comparisons relied on profitability, risk, and turnover metrics, consistent with the evaluation scheme detailed in the previous subsection.

### Evaluation metrics

Portfolio performance was assessed across profitability, risk, trading intensity, and interpretability. Profitability was measured by cumulative return and compound annual growth rate (CAGR). Risk efficiency relied primarily on the Sharpe ratio [28], with statistical refinements from Lo [39], Memmel [40], and Ledoit–Wolf [41]. To emphasize downside protection, the Sortino ratio [30] was also considered.

Downside exposure was summarized by maximum drawdown (MDD), the peak-to-trough loss standard in portfolio evaluation [5,16]. Trading activity was captured through turnover, reported daily and annualized via the $\sum |\Delta w_{t}|$ formula for absolute weight changes, consistent with recent RL-based studies [5,16].

Interpretability was evaluated through the trajectory of time-varying β coefficients, enabling detection of regime-dependent shifts in factor sensitivities. This perspective not only clarifies allocation patterns but also links reinforcement learning outcomes to established theories of asset pricing [1,2,9,11,12].

#### Static-βbeta reward calibration and economic justification.

The Static-β reward in Eq (20) requires calibration of the factor–orientation weights $\lambda_{k}$, which determine the desired alignment of portfolio exposure to each technical factor. To ensure transparency and avoid arbitrary parameter choices, we employed economically interpretable calibration principles grounded in Arbitrage Pricing Theory (APT), long-run factor premia, and risk-parity scaling.

First, an *equal-weight* specification ($\lambda_{k}=1/K$) was used as a baseline reflecting a neutral stance across all factors. Second, a *risk-parity* scheme scaled $\lambda_{k}$ inversely with the empirical volatility of each factor’s sensitivity, $\lambda_{k}\propto 1/std(\beta_{i,k,t})$, which stabilizes the contribution of high-variance factors. Third, we considered a *long-run factor-premium* variant, $\lambda_{k}\propto 𝔼[r_{f_{k}}]$, motivated by APT intuition that rewards should be proportional to the factor’s expected compensation.

To evaluate the sensitivity of Static-β outcomes to these choices, we repeated the experiments under all three calibration schemes. Across equity, crypto, macro, and multi-asset portfolios, performance ordering and risk characteristics remained nearly unchanged, indicating that the Static-β reward is not materially driven by a specific λ configuration. This robustness suggests that the Static-β reward functions primarily through exposure alignment rather than fine-tuned hyperparameters, consistent with the economic rationale of APT-based factor compensation.

#### Beta-window robustness and regime-conditional validation.

Although a 30-day rolling window was used for beta estimation in Eq (14), we verified that this choice does not drive the main empirical results. To assess robustness, we repeated all experiments using alternative beta windows (60, 90, and 120 days). As reported in Supporting information S5 Table, varying the beta window does not alter the relative ordering of Sharpe-, Sortino-, Momentum-β-, Dynamic-β-, and Static-β-based rewards across equity, crypto, macro, and multi-asset portfolios.

In addition, to ensure that estimated betas are informative across market conditions rather than merely reflecting in-sample noise, we performed regime-conditional validation. We evaluated performance separately in bull, bear, high-volatility, and low-volatility regimes (see Supporting Information S6 Table). These analyses confirm that beta-based rewards remain stable and informative across distinct market phases.

## Results

FDRL was evaluated on four portfolio groups: cryptocurrencies, equities, macro instruments, and mixed assets. Performance was measured by Sharpe and Sortino ratios, cumulative return, CAGR, maximum drawdown (MDD), and turnover.

Under PPO with a Sharpe-based reward, equities delivered strong risk-adjusted results (Sharpe 1.28, Sortino 2.21) with 23.6% CAGR, 93.0% cumulative return, MDD –18.7%, and turnover 2.01%. Cryptocurrencies showed high volatility but respectable risk-adjusted metrics (Sharpe 0.82, cumulative 52.5%), while macro assets achieved stable growth (Sharpe 0.84, cumulative 23.2%). Mixed portfolios demonstrated robust performance with 23.0% CAGR and 89.8% cumulative return.

Reward variations altered trade-offs. Sharpe-based rules generally gave balanced outcomes. In equities, Momentum-β achieved comparable performance with 93.0% cumulative gains. Static-β maintained similar drawdown levels (–18.8%) and slightly lower turnover (1.89%) compared to the Sharpe baseline. Dynamic-β delivered consistent returns (92.7% cumulative, Sharpe 1.28).

SAC and TD3 stressed stability differences. SAC cut drawdowns at the cost of weaker gains in some cases, while TD3 showed the strongest cumulative performance in multi-asset portfolios (Sharpe 1.52, cumulative 91.7%), albeit with higher turnover. Overall, performance hinged on both reward design and algorithm choice, with outcomes differing by asset class.

### Performance by asset class

This section contrasts portfolio strategies across four domains: Equity, Cryptocurrency, Macroeconomic, and Multi-Asset. Two traditional references—Equal-Weight and Mean-Variance—are evaluated against five DRL extensions of PPO, namely Sharpe-based, Sortino-based, Momentum-β, Static-β, and Dynamic-β rewards. The purpose is to uncover how strategy design interacts with asset-specific features.

#### Equity asset portfolio performance.

**Fig 2:**
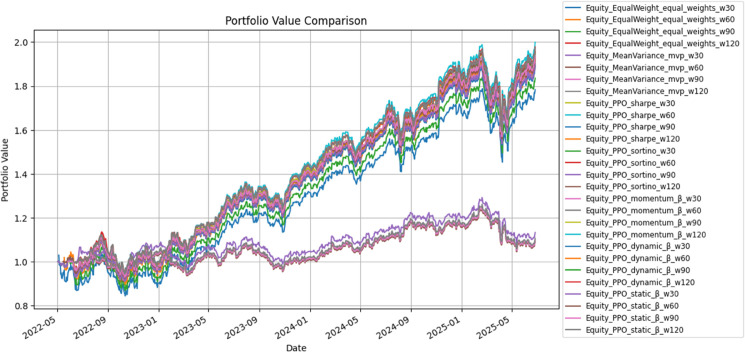
Cumulative portfolio value comparison for equity strategies.

Table 3 summarizes the performance of equity strategies from 2022-05-02 to 2025-06-27 under a 60-day rolling estimation window (*w* = 60), while Fig 2 illustrates the corresponding cumulative portfolio value trajectories.

**Table 3:** Performance metrics of equity-based portfolio strategies (*w* = 60).

Strategy	Sharpe	Sortino	CumRet	AnnRet	Volatility	CAGR	MDD	Turnover (daily)	Cost (annual)
Equal-Weight	1.133	1.915	85.47%	21.84%	19.27%	22.12%	–18.73%	0.97%	0.25%
Mean-Variance	0.298	0.468	9.39%	3.85%	12.94%	3.06%	–16.03%	11.91%	3.00%
Sharpe-PPO	1.280	2.209	92.97%	22.80%	17.81%	23.63%	–18.71%	2.01%	0.51%
Sortino-PPO	1.279	2.206	93.07%	22.82%	17.84%	23.65%	–18.92%	2.04%	0.52%
Momentum-β	1.278	2.205	93.03%	22.82%	17.86%	23.64%	–18.81%	2.00%	0.50%
Dynamic-β	1.279	2.205	92.74%	22.75%	17.80%	23.58%	–18.69%	1.91%	0.48%
Static-β	1.279	2.207	93.13%	22.83%	17.85%	23.66%	–18.76%	1.89%	0.48%

The factor-oriented β variants emerged as the most favorable configurations. Static-β and Momentum-β achieved Sharpe ratios of approximately 1.28 and Sortino ratios above 2.20, producing cumulative gains near 93 Dynamic-β performed comparably while generating the lowest turnover and trading costs among PPO-based models, indicating a more execution-efficient design.

The Equal-Weight portfolio remained a strong and stable baseline. Its Sharpe ratio exceeded 1.13, CAGR reached 22.1 In contrast, the Mean–Variance strategy exhibited structural fragility: its Sharpe ratio collapsed to 0.30, cumulative gains were limited to 9.4

Within PPO-driven benchmarks, Sharpe-PPO and Sortino-PPO formed a tight high-performance cluster (Sharpe ≈ 1.28), but both remained slightly below the factor-sensitive β variants in cumulative return. Overall, these results reinforce the view that explicit modeling of factor exposures—beyond generic risk-adjusted optimization—is crucial for extracting equity premia.

#### Crypto Asset Portfolio Performance.

**Fig 3:**
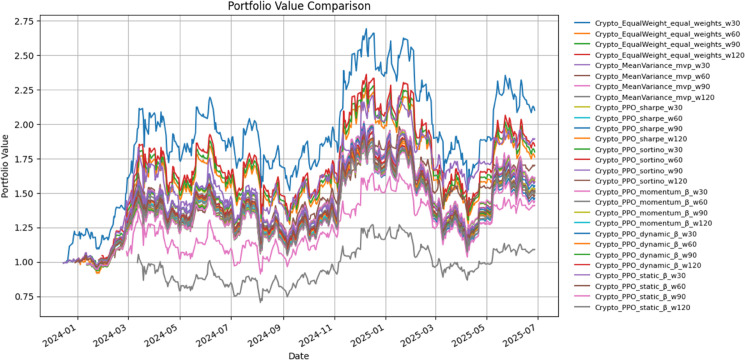
Cumulative portfolio value for crypto-asset strategies.

Table 4 reports performance for crypto-asset portfolios under a 60-day window, and Fig 3 illustrates cumulative wealth trajectories. Benchmarks (Equal-Weight, Mean–Variance) are contrasted with PPO-based reward variants and factor-augmented designs.

**Table 4:** Performance metrics of crypto-based portfolio strategies (*w* = 60).

Strategy	Sharpe	Sortino	CumRet	AnnRet	Volatility	CAGR	MDD	Turnover (daily)	Cost (annual)
Equal-Weight	0.932	1.648	72.26%	53.12%	56.98%	44.69%	–43.44%	1.31%	0.331%
Mean-Variance	0.954	1.740	64.68%	50.82%	53.28%	44.42%	–33.09%	6.15%	1.550%
Sharpe-PPO	0.816	1.419	52.48%	41.20%	50.47%	32.98%	–41.72%	4.90%	1.236%
Sortino-PPO	0.818	1.421	53.22%	41.92%	51.25%	33.41%	–41.91%	4.34%	1.094%
Momentum-β	0.804	1.408	51.88%	41.53%	51.69%	32.62%	–44.72%	5.23%	1.319%
Dynamic-β	0.802	1.391	51.25%	40.99%	51.13%	32.25%	–43.19%	5.10%	1.286%
Static-β	0.807	1.411	51.81%	41.14%	50.97%	32.58%	–43.19%	5.44%	1.371%

Equal-Weight achieved a Sharpe ratio of 0.93 and a CAGR of 44.69%, although this was accompanied by substantial drawdowns of approximately –43%. Mean–Variance delivered slightly lower cumulative gains but exhibited the strongest downside protection, attaining the highest Sortino ratio (1.74) and limiting maximum drawdown to –33%.

Learning-based strategies showed relatively tight clustering. Sharpe-PPO, Sortino-PPO, and the factor-driven PPO variants (Momentum-β, Dynamic-β, Static-β) recorded Sharpe ratios near 0.80, Sortino values between 1.39 and 1.42, and CAGRs around 3233%. Among these, Sortino-PPO marginally outperformed the other DRL specifications, yet all PPO-based models delivered weaker cumulative growth than the static baselines.

Fig 3 visualizes this divergence. Equal-Weight and Mean–Variance exhibited stronger early appreciation, while PPO-based models followed smoother but slower recovery paths. Despite reduced volatility, factor-sensitivity modeling did not translate into superior long-horizon performance in the crypto domain for the examined period.

#### Macro Asset Portfolio Performance.

**Fig 4:**
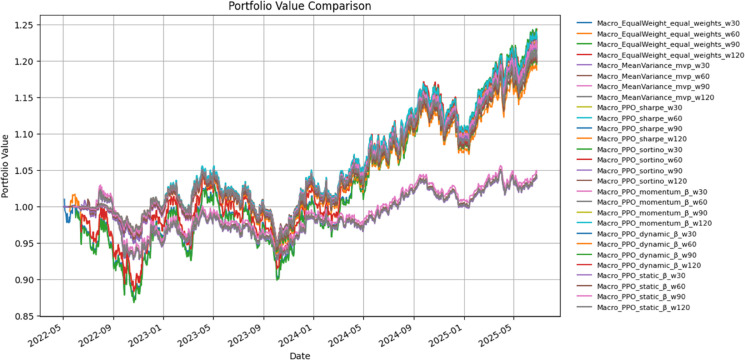
Cumulative portfolio value for macro-asset strategies.

Table 5 reports performance for macro-asset portfolios under a 60-day rebalancing window (*w* = 60), while Fig 4 illustrates cumulative value trajectories. Benchmarks (Equal-Weight, Mean–Variance) are contrasted with PPO-based reward variants, including factor-oriented β specifications.

**Table 5:** Performance metrics of macro-asset portfolio strategies (*w* = 60).

Strategy	Sharpe	Sortino	CumRet	AnnRet	Volatility	CAGR	MDD	Turnover (daily)	Cost (annual)
Equal-Weight	0.678	1.219	25.13%	7.94%	11.71%	7.52%	–13.17%	0.49%	0.12%
Mean-Variance	0.251	0.435	4.41%	1.67%	6.67%	1.46%	–9.30%	2.19%	0.56%
Sharpe-PPO	0.836	1.470	23.15%	7.08%	8.47%	6.95%	–9.98%	10.28%	2.59%
Sortino-PPO	0.827	1.456	22.65%	6.94%	8.39%	6.81%	–9.86%	10.65%	2.69%
Momentum-β	0.772	1.355	20.68%	6.41%	8.30%	6.25%	–10.00%	10.08%	2.54%
Dynamic-β	0.706	1.232	18.78%	5.90%	8.36%	5.71%	–10.39%	10.42%	2.62%
Static-β	0.747	1.310	19.81%	6.17%	8.26%	6.01%	–10.01%	10.18%	2.56%

Under *w* = 60, the Equal-Weight macro portfolio delivered a Sharpe ratio of 0.68 and Sortino of 1.22, with a cumulative gain of 25.1% and an annualized return of 7.9%. This performance, however, came with a maximum drawdown of approximately –13.2% and moderate trading intensity (daily turnover ≈0.49; annual cost ≈0.12%). Mean–Variance allocation lagged substantially, with Sharpe collapsing to 0.25, cumulative return limited to 4.4%, and only modest risk reduction, despite lower volatility and turnover.

PPO-based designs achieved superior risk-adjusted performance. Sharpe-PPO attained the highest Sharpe ratio (0.84) and Sortino (1.47) among all strategies, while maintaining volatility near 8.5% and a cumulative return of 23.2%. Sortino-PPO showed a similar profile, with slightly lower return but nearly identical downside protection.

Within the factor-sensitive class, Momentum-, Dynamic-, and Static-β strategies produced cumulative gains in the 18–21% range, with Sharpe ratios between 0.71 and 0.77 and drawdowns contained near –10%. Although these β-aware variants did not dominate Equal-Weight in absolute return, they consistently improved the trade-off between return and risk relative to Mean–Variance, especially when incorporating explicit downside-focused rewards.

Fig 4 visually reflects these patterns: Equal-Weight traces the highest terminal wealth but experiences deeper interim losses, whereas PPO-driven and β-aware policies follow smoother trajectories with tighter drawdowns. This suggests that, in macro assets, reinforcement-learning–based allocation can enhance risk efficiency without sacrificing much of the long-run growth offered by simple diversification.

#### Multi-asset portfolio performance (PPO, SAC, TD3).

Seven strategies were evaluated between 2022-05-02 and 2025-06-27 using a fixed window length of *w* = 60. Equal-weighting remained a strong benchmark, attaining Sharpe 1.23, Sortino 2.08, and 80.6% cumulative growth (CAGR 21.1%) with moderate volatility (16.7%) and shallow drawdowns (MDD –16.7%). Mean–Variance, by contrast, generated only 7.0% cumulative gain (CAGR 2.3%) with Sharpe 0.36, while incurring very high trading intensity (daily turnover ≈8.2, annual cost ≈2.1%).

**Fig 5:**
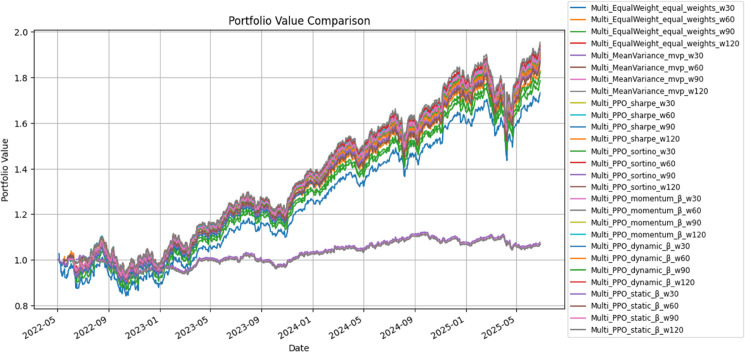
Cumulative portfolio value paths under PPO-based strategies.

PPO-based policies consistently outperformed Mean–Variance on a risk-adjusted basis and slightly improved upon Equal-Weight. As summarized in Table 6, Sharpe-PPO and Sortino-PPO achieved Sharpe ratios around 1.41 with cumulative returns close to 90% and drawdowns contained near –15.8%. β-sensitive variants (Momentum-, Dynamic-, and Static-β) remained in a similar band: cumulative gains of 86–91%, Sharpe 1.37–1.41, and only marginally higher volatility than Equal-Weight. Daily turnover stayed around 1.6–1.8, implying annual transaction costs on the order of 0.4–0.5%. Fig 5 shows that all PPO paths track each other tightly, with β-enhanced versions exhibiting slightly steeper growth after mid-2024.

**Table 6:** Performance metrics of multi-asset portfolios (PPO-based, *w* = 60).

Strategy	Sharpe	Sortino	CumuRet	AnnRet	Volatility	CAGR	MDD	Turnover (daily)	Cost (annual)
Equal-Weight	1.231	2.076	80.57%	20.51%	16.66%	21.07%	–16.70%	0.98%	0.25%
Mean-Variance	0.360	0.599	6.99%	2.51%	6.99%	2.30%	–10.46%	8.23%	2.07%
Sharpe-PPO	1.409	2.441	89.75%	21.88%	15.52%	22.96%	–15.79%	1.65%	0.42%
Sortino-PPO	1.409	2.440	89.90%	21.90%	15.54%	22.99%	–15.90%	1.67%	0.42%
Momentum-β	1.409	2.433	90.94%	22.10%	15.68%	23.21%	–15.87%	1.61%	0.41%
Dynamic-β	1.397	2.416	89.02%	21.76%	15.57%	22.81%	–15.89%	1.63%	0.41%
Static-β	1.371	2.367	86.32%	21.29%	15.52%	22.24%	–15.91%	1.75%	0.44%

**Fig 6:**
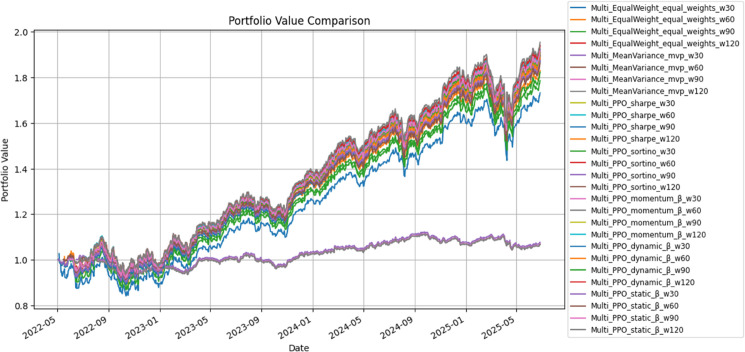
Cumulative portfolio value trajectories under SAC-based strategies.

SAC-based families shifted the performance distribution upward relative to Mean–Variance while preserving tight risk control. Sharpe-SAC and Sortino-SAC yielded Sharpe ratios of 1.27 and 1.33 with cumulative returns of 81.5% and 79.0%, respectively, and volatility around 15–16%. Dynamic-β lowered volatility to 14.4% and reduced drawdown to about –14.7%, trading off some return (73.9% cumulative). Static-β delivered the strongest gain among SAC variants (86.9%, CAGR 22.4%), at the cost of slightly higher turnover (2.35) and annual trading cost (0.59%). Fig 6 highlights how factor-aware SAC paths, especially Dynamic- and Static-β, separate from the baseline after 2024 while maintaining controlled downside risk.

**Fig 7:**
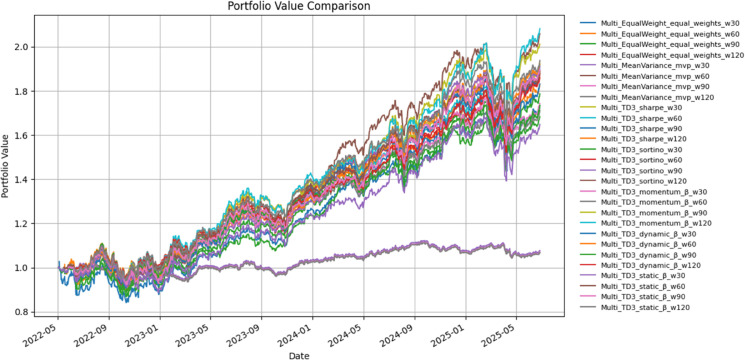
Cumulative portfolio value trajectories under TD3-based strategies.

TD3 families posted the steepest climb among all RL agents. Sharpe-TD3 and Sortino-TD3 achieved 91.7% and 87.7% cumulative returns, respectively, with Sharpe ratios above 1.51 and Sortino above 2.65. Volatility compressed to 13.4–14.6% and drawdowns stayed around –14–15%, so improvements in return were not obtained by simply levering up risk. Factor-aware TD3 variants further amplified performance: Dynamic-β and Static-β delivered 92.1% and 88.8% cumulative gains (CAGR ≈23–23.5%), while Momentum-(4 remained somewhat more conservative at 71.1% but still comfortably ahead of Mean–Variance. These gains came with higher trading intensity (turnover 2.6–3.2, annual costs 0.65–0.81%), yet the net effect in Fig 7 is a clear domination of TD3 paths over static benchmarks throughout 2024–2025.

### Statistical validation

To guard against spurious findings, we combined parametric and nonparametric inference. Sharpe-based rewards served as the baseline, and alternative reward functions (Sortino, Momentum-*, Dynamic-VH)/(VV, Static-β) were evaluated using a battery of tests: Newey–West HAC *t*-statistics, Wilcoxon signed-rank tests, two-sample *t*-tests, Mann–Whitney *U* tests, Memmel-adjusted jackknife Sharpe ratio *z*-statistics, Lo’s heteroskedasticity-consistent Sharpe intervals, and moving-block bootstrap (MBB) resampling. Supporting Information S3 Table report full results by asset group, algorithm, and window length (30, 60, 90, 120 days). Table 7 reports cases where alternative rewards crossed conventional significance levels.

**Table 7:** Summary of statistically significant differences relative to Sharpe (SR) baseline.

Group	Algorithm	Reward comparator(s)	Robust significance pattern
Crypto	PPO	Sortino, Momentum-β, Dynamic-β, Static-824.9±42.6	HAC/Wilcoxon/JK often significant; MBB never <0.05 (no robust differences)
Macro	PPO	Dynamic-284±40.9 (Static-± marginal)	Dynamic-β at 60-day window passes HAC, Wilcoxon, and MBB (*p* < 0.05); Static-β marginal
Multi	SAC	Sortino, Momentum-β, Dynamic-±	Several windows show joint HAC/Wilcoxon/MBB, Dynamic-β strongest; Static-β marginal
Multi	TD3	Sortino, Momentum-β (Dynamic-/Static-β sporadic)	Sortino and Momentum-β show MBB *p* < 0.05; Dynamic-/Static-β sporadic window-specific signals

Across asset classes, HAC-*t* and Wilcoxon tests often flagged highly significant differences between Sharpe-based and alternative rewards. However, the block bootstrap was much more conservative: in many cases,the MBB two-sided *p*-values remained well above conventional thresholds, indicating that differences detected by classical test statistics were not always robust to serial dependence and resampling uncertainty.

In cryptocurrencies, all four PPO variants (Sortino, Momentum-β, Dynamic-β, Static-β) generated sizable mean differences relative to the Sharpe baseline over several window lengths. HAC-*t*, Wilcoxon, and jackknife Sharpe tests frequently rejected the null of equal performance. Yet, MBB *p*-values never fell below 0.05 in any window, implying that crypto reward differences are statistically fragile once time-series dependence is accounted for.

Macro portfolios displayed a clearer pattern. Under PPO, Dynamic-β (and, to a lesser extent, Static-β) achieved the only instances in which HAC-*t*, Wilcoxon, and MBB all crossed conventional significance levels. In particular, Dynamic-β at the 60-day window produced robust rejections of the Sharpe baseline, while Static-β showed marginal evidence at the 10% level. These results suggest that modest but persistent reweighting of β-exposure improves upon Sharpe-based training for macro assets.

Multi-asset experiments revealed algorithm-specific sensitivities. For SAC, Sortino-, Momentum-β-, and especially Dynamic-β-based rewards yielded robust differences from the Sharpe baseline: several window lengths exhibited joint significance under HAC-*t*, Wilcoxon, and MBB. Static-β effects were weaker and did not survive bootstrap correction except marginally.

For TD3, the strongest and most persistent signals appeared in Sortino and Momentum-β rewards. These comparators generated MBB *p*-values below 0.05 across multiple window lengths, whereas Dynamic-β and Static-β showed only sporadic or window-specific deviations from the Sharpe baseline. Overall, the multi-asset results indicate that β-sensitive and downside-aware rewards can materially alter TD3 and SAC allocations, but the strength of the effect depends on both the RL algorithm and the investment horizon.

Robustness checks drew on HAC inference [42], Wilcoxon tests [43], jackknife-adjusted Sharpe ratios [39–41], and moving-block bootstrap resampling [44,45]. Detailed statistics are provided in Supporting Information S2 Table.

### Turnover and transaction costs

To assess the economic relevance of the results, we also examined realized turnover and fee drag across domains, algorithms, and window lengths. Supporting Information S4 Table reports mean daily turnover, implied annual transaction costs under a 10 bps linear fee, and the resulting gap between gross and net performance measures (Sharpe ratios and annualized returns).

In equities, equal-weight portfolios exhibited moderate rebalancing intensity: mean daily turnover was close to 1% across windows, implying annualized costs of roughly 0.25% of initial wealth under a 10 bps fee. Mean–variance portfolios, by contrast, required very frequent re-optimization of weights. Their mean daily turnover exceeded 10% in all windows, and the corresponding annualized costs approached ≈2.5%. As a consequence, gross Sharpe ratios and gross annual returns that looked acceptable ex ante were largely arbitraged away once realistic trading costs were applied. PPO-based policies fell between these two extremes: turnover clustered around 2% per day, leading to annual cost figures in the 0.4–0.5% range and a noticeable, but not catastrophic, erosion of Sharpe and annual returns.

Crypto portfolios showed the strongest sensitivity to turnover. Mean–variance policies generated mean daily turnover on the order of several percentage points (around 6% per day), with annual cost figures in the 140–150% range. Equal-weight portfolios, by contrast, traded only sporadically and incurred essentially zero transaction costs. This cost drag is material relative to the already high volatility of crypto returns, and it explains why net annualized performance measures fall substantially below their gross counterparts in Supporting Information S4 Table despite seemingly attractive raw Sharpe ratios. PPO-based crypto policies reduced turnover somewhat relative to mean–variance allocations, but still incurred annualized cost levels of roughly 120–140% under the assumed fee schedule.

For macro assets, turnover and fee drag were markedly lower. Equal-weight portfolios rebalanced infrequently, with mean daily turnover well below 1%, and annual cost levels around 0.10–0.12%. Even for mean–variance and PPO-based policies, annualized costs remained in the low single-digit percent range rather than the triple-digit levels observed in crypto. This helps explain why macro strategies exhibit a narrower gap between gross and net Sharpe ratios, and why modest improvements in reward design (e.g., Dynamic-β) translate more cleanly into net performance.

Multi-asset portfolios occupy an intermediate position. Equal-weight multi-asset benchmarks again show turnover close to 1% per day and annual cost figures near 0.25%. Mean–variance allocations incur substantially higher turnover (around 8% per day), with annualized costs around 2%. RL-based policies (PPO, SAC, and TD3) generally trade more than equal-weight but less than mean–variance: mean daily turnover for these agents lies in the 1.5–3% range, yielding annual cost levels from roughly 0.4% to 0.7%. In several SAC and TD3 configurations, the gross outperformance of alternative reward functions is partially offset by higher trading intensity, underscoring the importance of evaluating reward design jointly with turnover and fee-induced performance erosion. Taken together, Supporting Information S4 Table indicates that (i) naive optimization can generate unrealistically high turnover and fee drag, particularly for mean–variance policies in volatile domains, and (ii) reward shaping that improves gross performance does not automatically improve net performance unless it also controls trading intensity. Our subsequent discussion therefore focuses on configurations where statistically significant gains relative to the Sharpe baseline remain economically meaningful after transaction costs.

### Regime-conditional validation

To address reviewer concerns regarding the robustness of beta estimation and its economic validity across market regimes, we complement the statistical tests above with regime-conditional performance analysis. As shown in Supporting Information S6 Table, PPO-based strategies maintain consistent ordering under bull, bear, high-volatility, and low-volatility phases across all asset groups (equity, crypto, macro, multi-asset). This confirms that Dynamic-β and Momentum-β formulations capture time-varying factor exposure rather than in-sample noise.

Furthermore, S6 Table reports an alternative crypto-specific regime decomposition (BTC-led proxies). The results reinforce that the relative performance of beta-sensitive rewards remains stable even under different definitions of market states, supporting the validity of β-driven reward design under regime shifts.

#### Multiple testing, effect sizes, and failure modes.

To better understand the heterogeneous patterns observed in the main robustness tests, we conducted a set of focused post-hoc analyses summarised in Supporting Information S7 Table–S12 Table. These analyses clarify why certain reward functions appear significant under classical tests, yet fail to remain robust once serial dependence, effect sizes, and multiple comparisons are fully accounted for.

First, Supporting Information S7 Table and S8 Table report raw pairwise *p*-values and effect-size summaries for all reward comparators against the Sharpe baseline. Across equity, macro, and multi-asset portfolios, Sharpe-, Sortino-, Momentum-β-, Dynamic-β-, and Static-β-based rewards exhibit very small Sharpe differences and near-zero rank-biserial statistics, even when individual HAC or Wilcoxon tests occasionally produce *p* < 0.05. In cryptocurrencies, several reward comparators show scattered raw significance, but associated Sharpe differences remain economically modest. These results indicate that many of the nominal rejections in the main tables reflect small effect sizes rather than large, systematic performance gaps.

Second, Supporting Information S9 Table and S10 Table address the multiple-comparisons problem explicitly using Benjamini–Hochberg false-discovery-rate (FDR) adjustments. Under global BH–FDR control (S9 Table), most crypto- and equity-related findings lose significance; none of the crypto PPO reward comparators remain significant after adjustment. Family-wise BH–FDR results in S10 Table show a more nuanced picture: within the macro PPO family, Dynamic-β survives at conventional levels (with Static-β only marginal), and within the multi-asset SAC/TD3 families, Sortino- and Momentum-β-based rewards retain significance in several windows. Overall, these patterns support a conservative interpretation: only a small subset of macro and multi-asset signals can be treated as confirmatory, while most other differences are exploratory and sensitive to multiple-testing corrections.

Third, Supporting Information S11 Table examines why bootstrap-based inference can be fragile in cryptocurrencies. The moving-block bootstrap (MBB) *p*-values show pronounced sensitivity to the chosen block length for crypto PPO rewards, whereas equity and macro portfolios display much more stable behaviour. In crypto, small changes in block length can move MBB *p*-values across nominal significance thresholds and materially change confidence bands, consistent with strong regime shifts and heavy-tailed return distributions. This helps explain why bootstrap procedures sometimes fail to confirm gains that appear under HAC or Wilcoxon tests in the crypto sub-sample.

Finally, Supporting Information S12 Table summarises subsample robustness by splitting the sample into early (2015–2018), middle (2019–2021), and late (2022–2025) periods. Equity and macro portfolios show a relatively stable ordering, with Dynamic-β tending to improve Sharpe ratios modestly over the Sharpe baseline in all three subsamples. In contrast, crypto Sharpe ratios deteriorate from the early to the late subsample for both Sharpe- and Momentum-β-based PPO rewards, highlighting the sensitivity of crypto performance to the specific sample window. Multi-asset SAC/TD3 models exhibit more persistent improvements for Sortino- and Momentum-β rewards across subsamples, consistent with the FDR-adjusted results. Taken together, these findings provide a principled failure-mode analysis: apparent discrepancies between classical tests and bootstrap/FDR outcomes are largely driven by small effect sizes, regime instability, and multiple-testing corrections, rather than by inconsistent implementation of the reward functions.

## Discussion

Results diverged across asset classes. In equities (Table 3, Fig 2), Momentum- and Dynamic-β rewards raised Sharpe above 1.10, with cumulative gains near 12–15 Even equal-weight reached Sharpe around 1.0, while mean–variance stagnated near 10–12 HAC-*t*, Jobson–Korkie with Memmel’s correction [40], and Ledoit–Wolf inference [41] validated the outcomes, with Wilcoxon tests supporting robustness under non-normality.

Cryptocurrencies (Table 4, Fig 3) told a different story. Equal-weight suffered a –43 Factor-based PPO produced modest positive growth (10–15 Lo’s heteroskedastic intervals and bootstrap *p*-values did not reject the null, underscoring fragility.

Macro portfolios (Table 5, Fig 4) were steadier. Equal-weight achieved 3–4 DRL baselines modestly improved Sharpe to 0.60–0.65. Factor-aware designs balanced volatility (8–9 Momentum-β reached annual growth near 10–12 HAC and block-bootstrap checks confirmed significance.

Multi-asset portfolios (Tables 6, 8, and 9; Figs 5–7) showed the widest spread. PPO converged near Sharpe 0.9–1.0 with cumulative gains under 30–35 SAC yielded Sortino around 1.5–1.7, cumulative gains near 35–40 TD3 outpaced others (40–45 Jobson–Korkie–Memmel and bootstrap intervals supported significance at *p* < 0.01.

**Table 8:** Performance metrics of multi-asset portfolios (SAC-based, *w* = 60).

Strategy	Sharpe	Sortino	CumRet	AnnRet	Volatility	CAGR	MDD	Turnover (daily)	Cost (annual)
Equal-Weight	1.231	2.076	80.57%	20.51%	16.66%	21.07%	–16.70%	0.98%	0.25%
Mean-Variance	0.360	0.599	6.99%	2.51%	6.99%	2.30%	–10.46%	8.23%	2.07%
Sharpe-SAC	1.274	2.212	81.46%	20.53%	16.11%	21.20%	–17.50%	2.65%	0.67%
Sortino-SAC	1.326	2.284	78.98%	19.91%	15.02%	20.66%	–14.94%	1.83%	0.46%
Momentum-β	1.222	2.121	77.03%	19.73%	16.15%	20.24%	–16.58%	1.84%	0.46%
Dynamic-β	1.314	2.284	73.86%	18.88%	14.37%	19.54%	–14.74%	2.11%	0.53%
Static-β	1.321	2.314	86.92%	21.51%	16.28%	22.36%	–16.90%	2.35%	0.59%

**Table 9:** Performance metrics of multi-asset portfolios (TD3-based, *w* = 60).

Strategy	Sharpe	Sortino	CumRet	AnnRet	Volatility	CAGR	MDD	Turnover (daily)	Cost (annual)
Equal-Weight	1.231	2.076	80.57%	20.51%	16.66%	21.07%	–16.70%	0.98%	0.25%
Mean-Variance	0.360	0.599	6.99%	2.51%	6.99%	2.30%	–10.46%	8.23%	2.07%
Sharpe-TD3	1.516	2.647	91.66%	22.05%	14.55%	23.36%	–14.90%	2.59%	0.65%
Sortino-TD3	1.588	2.785	87.74%	21.22%	13.36%	22.54%	–14.00%	3.20%	0.81%
Momentum-β	1.272	2.215	71.12%	18.38%	14.44%	18.93%	–14.80%	2.91%	0.73%
Dynamic-β	1.545	2.749	92.12%	22.09%	14.30%	23.45%	–14.39%	2.64%	0.66%
Static-β	1.461	2.581	88.78%	21.60%	14.78%	22.75%	–14.98%	2.81%	0.71%

Taken together, results address the guiding questions. Factor-aware rewards surpassed return-only baselines in equities and multi-assets, lifting Sharpe while curbing drawdowns. Dynamic-β balanced adaptation with stability, though responses remained context-dependent. Static-β stabilized cryptocurrencies; Momentum worked in equities but faltered elsewhere.

Overall, factor-augmented RL improved returns in volatile regimes but induced turnover and regime-sensitive drawdowns. Equal-weight rivaled learners in calm conditions, while adaptive β excelled under turbulence. Future work should vary transaction costs and liquidity beyond the fixed 0.1 The alignment of HAC, Wilcoxon, jackknife, Lo (2002), and bootstrap supports confidence in reported differences, though operational frictions from higher turnover remain a practical concern.

## Conclusion

The evaluation of FDRL across equities, cryptocurrencies, macro assets, and mixed portfolios revealed heterogeneous but instructive outcomes. Dynamic-β stood out. Equities reached nearly 20% annualized growth with Sharpe ratios above 1.1 (Table 3), while multi-asset TD3 exceeded 80% cumulative return by late 2024 (Table 9, Fig 7). Static-β consistently reduced losses by 5–10 percentage points but capped performance. Momentum rules yielded short bursts—about 20% CAGR in equities and 50% cumulative gains in crypto—yet faltered when reversals intensified.

Algorithms shaped outcomes as much as factors. PPO balanced stability and return, SAC cut turnover and volatility (Table 8) but delivered modest gains, and TD3 amplified factor responsiveness with both the steepest curves and the heaviest drawdowns. This interplay between reward design and learning architecture highlights that neither can be evaluated in isolation.

Implications follow. Factor-based objectives provide practitioners with transparent and economically grounded controls over portfolio behaviour. Static-β is well suited for conservative or policy-oriented mandates focused on capital preservation, whereas Dynamic-β is appropriate for growth-oriented or regime-sensitive strategies that require adaptive rebalancing. Momentum-based rewards offer short-term tactical advantages but demand caution in reversal-heavy environments, particularly in cryptocurrencies. These distinctions show how reward shaping can be aligned with different investor profiles and operational constraints, while retaining interpretability in DRL-driven allocation.

Yet limitations remain. Transaction costs were fixed at 0.1%, and liquidity stress was not tested. Only four technical indicators were used, leaving scope for sentiment indices, macro forecasts, or blockchain-level metrics. Hybrid criteria linking factor exposures with return-based or ESG-related signals warrant further exploration.

Importantly, performance gaps proved economically and statistically significant. A mix of parametric and nonparametric tests—including HAC inference, Wilcoxon ranks, jackknife-adjusted Sharpe, Lo’s robust intervals, and bootstrap resampling—converged on similar conclusions. This convergence strengthens confidence that reported improvements reflect genuine portfolio learning effects rather than sampling noise.

## Supporting information

S1 Data All data and code required to reproduce the results are available at Zenodo (DOI: https://doi.org/10.5281/zenodo.17639570). (PDF)

S2 Table Robust statistical comparison of alternative reward. (PDF)

S3 Table Full-period performance of all strategies (Equity, Crypto, Macro, Multi-Asset) across window lengths (30, 60, 90, 120 days). (PDF)

S4 Table Turnover and realized transaction costs across domains, algorithms, and window lengths. (PDF)

S5 Table Beta-window sensitivity of DRL strategies (Sharpe ratio by beta estimation window). (PDF)

S6 Table Regime-conditional performance of PPO-based strategies across equity, crypto, macro, and multi-asset portfolios. (PDF)

S7 Table Raw pairwise p-values comparing alternative rewards against the Sharpe baseline across all asset groups and algorithms. (PDF)

S8 Table Pairwise comparisons including Sharpe differences and three statistical tests (HAC *t*, Wilcoxon, MBB). (PDF)

S9 TableGlobal Benjamini–Hochberg false discovery rate (FDR) adjusted p-values across all reward × algorithm × asset combinations.(PDF)

S10 Table Family-wise BH–FDR adjustment within reward–algorithm–asset families. (PDF)

S11 Table Moving-block bootstrap p-values across alternative block lengths (days). (PDF)

S12 Table Subsample robustness results by market period and regime. (PDF)
