# Supplementary material for: Factor-based deep reinforcement learning for asset allocation: Comparative analysis of static and dynamic beta reward designs
Source: PLoS One. 2025 Dec 30;20(12):e0332779. doi: 10.1371/journal.pone.0332779 (PMC12753089; doi:10.1371/journal.pone.0332779)
Supplement: S1 Data — (PDF) [file pone.0332779.s001.pdf]

S1 Data. Zenodo archive of data and code

This Supporting Information item documents the full structure of the Zenodo repository containing all data and code used to reproduce the results of the manuscript.

Zenodo DOI: https://doi.org/10.5281/zenodo.17639570

Repository Structure

```
nhjung-phd/FactorBasedDRL-v1.0.0.zip
  nhjung-phd-FactorBasedDRL-c4dbf2c
       Factor_based_DRL.ipynb
       README.md
       Result/
             Fig2.png
             Fig3.png
             Fig4.png
             Fig5.png
             Fig6.png
             Fig7.png
```

Contents Description

- **Factor_based_DRL.ipynb** — Notebook implementing all experiments.
- **README.md** — Instructions for environment setup and reproduction.
- **Result/** — Output figures reproduced in the manuscript.
