# Supplementary material for: Factor-based deep reinforcement learning for asset allocation: Comparative analysis of static and dynamic beta reward designs
Source: PLoS One. 2025 Dec 30;20(12):e0332779. doi: 10.1371/journal.pone.0332779 (PMC12753089; doi:10.1371/journal.pone.0332779)
Supplement: S2 Table — (PDF) [file pone.0332779.s002.pdf]

S2 Table. Robust statistical comparison of alternative reward functions vs. Sharpe (SR) baseline across varying window lengths (30, 60, 90, 120)

Group	Window	Algo	Comparator	N	Mean Diff (Ann.)	$t_{\mathrm{HAC}}(p)$	Wilcoxon p	t-test p	M-W Up	MBB p_{two}
Window = 30 days										
Equity	30	PPO	Sortino	791	1.37e+00	15.77*** (0.000)	0.000***	0.988	0.980	0.915
Equity	30	PPO	Momentum-β	791	1.05e+00	13.49*** (0.000)	0.000***	0.989	0.989	0.937
Equity	30	PPO	Dynamic-β	791	2.86e+00	16.55*** (0.000)	0.000***	0.974	0.965	0.793
Equity	30	PPO	Static-β	791	1.45e+00	13.23*** (0.000)	0.000***	0.982	0.971	0.881
Crypto	30	PPO	Sortino	383	-1.80e+00	-11.20 (1.000)	1.000	0.991	0.988	0.948
Crypto	30	PPO	Momentum-β	383	-3.62e+00	-15.26 (1.000)	1.000	0.985	0.969	0.944
Crypto	30	PPO	Dynamic-β	383	1.61e+00	2.45*** (0.007)	0.000***	0.987	0.977	0.952
Crypto	30	PPO	Static-β	383	8.45e-01	1.70** (0.045)	0.000***	0.997	0.973	0.899
Macro	30	PPO	Sortino	791	-3.10e-01	-8.49 (1.000)	1.000	0.986	0.991	0.790
Macro	30	PPO	Momentum-β	791	2.23e+00	13.22*** (0.000)	0.000***	0.927	0.945	0.218
Macro	30	PPO	Dynamic-β	791	1.01e+00	12.27*** (0.000)	0.000***	0.967	0.975	0.575
Macro	30	PPO	Static-β	791	1.38e+00	12.94*** (0.000)	0.000***	0.955	0.965	0.447
Multi	30	PPO	Sortino	791	7.76e+00	18.24*** (0.000)	0.000***	0.936	0.932	0.479
Multi	30	PPO	Momentum-β	791	-6.90e-01	-14.87 (1.000)	1.000	0.996	0.998	0.966
Multi	30	PPO	Dynamic-β	791	1.95e+00	60.43*** (0.000)	0.000***	0.991	0.992	0.945
Multi	30	PPO	Static-β	791	4.83e-01	4.81*** (0.000)	0.000***	0.994	0.993	0.860
Multi	30	SAC	Sortino	791	-1.25e+01	-10.01 (1.000)	1.000	0.761	0.671	0.007***
Multi	30	SAC	Momentum-β	791	-1.17e+01	-14.86 (1.000)	1.000	0.857	0.838	0.128
Multi	30	SAC	Dynamic-β	791	-1.95e+01	-13.00 (1.000)	1.000	0.792	0.737	0.005***
Multi	30	SAC	Static-β	791	-3.55e+00	-7.17 (1.000)	1.000	0.990	0.898	0.959
Multi	30	TD3	Sortino	791	2.56e+01	17.89*** (0.000)	0.000***	0.682	0.571	0.027**
Multi	30	TD3	Momentum-β	791	1.19e+01	10.06*** (0.000)	0.000***	0.791	0.708	0.020**
Multi	30	TD3	Dynamic-β	791	2.82e+00	11.84*** (0.000)	0.000***	0.956	0.937	0.666
Multi	30	TD3	Static-β	791	2.64e+01	15.32*** (0.000)	0.000***	0.699	0.649	0.003***
Window = 60 days										
Equity	60	PPO	Sortino	782	5.26e-01	9.58*** (0.000)	0.000***	0.999	0.995	0.969
Equity	60	PPO	Momentum-β	782	1.62e-01	5.69*** (0.000)	0.000***	0.999	0.996	0.977
Equity	60	PPO	Dynamic-β	782	9.89e-01	11.42*** (0.000)	0.000***	0.998	0.992	0.935
Equity	60	PPO	Static-β	782	3.09e-01	5.50*** (0.000)	0.000***	0.998	0.996	0.999
Crypto	60	PPO	Sortino	374	-2.34e+00	-14.52 (1.000)	1.000	0.990	0.992	0.920
Crypto	60	PPO	Momentum-β	374	-6.78e+00	-9.71 (1.000)	1.000	0.995	0.948	0.747
Crypto	60	PPO	Dynamic-β	374	-1.63e+00	-5.60 (1.000)	1.000	0.997	0.988	0.856
Crypto	60	PPO	Static-β	374	-4.45e+00	-11.19 (1.000)	1.000	0.999	0.995	0.830
Macro	60	PPO	Sortino	782	4.70e-01	12.12*** (0.000)	0.000***	0.984	0.985	0.747
Macro	60	PPO	Momentum-β	782	2.41e+00	13.17*** (0.000)	0.000***	0.921	0.956	0.171
Macro	60	PPO	Dynamic-β	782	4.16e+00	13.28*** (0.000)	0.000***	0.862	0.921	0.030**
Macro	60	PPO	Static-β	782	3.22e+00	13.26*** (0.000)	0.000***	0.893	0.927	0.076*
Multi	60	PPO	Sortino	782	-6.74e-01	-16.77 (1.000)	1.000	0.998	0.996	0.981
Multi	60	PPO	Momentum-β	782	-7.08e-01	-8.16 (1.000)	1.000	0.986	0.972	0.910
Multi	60	PPO	Dynamic-β	782	5.86e-01	8.03*** (0.000)	0.000***	0.993	0.991	0.883
Multi	60	PPO	Static-β	782	4.50e+00	14.03*** (0.000)	0.000***	0.962	0.960	0.551
Multi	60	SAC	Sortino	782	-1.01e+01	-12.56 (1.000)	1.000	0.961	0.884	0.085*
Multi	60	SAC	Momentum-β	782	-9.42e-01	-1.60 (0.945)	1.000	0.951	0.956	0.174
Multi	60	SAC	Dynamic-β	782	1.20e+00	1.21 (0.113)	0.958	0.893	0.821	0.058*
Multi	60	SAC	Static-β	782	-1.35e+01	-14.49 (1.000)	1.000	0.940	0.941	0.300
Multi	60	TD3	Sortino	782	3.12e+00	7.76*** (0.000)	0.000***	0.941	0.898	0.715
Multi	60	TD3	Momentum-β	782	9.66e+00	7.31*** (0.000)	0.000***	0.752	0.693	0.002***
Multi	60	TD3	Dynamic-β	782	-2.11e+00	-3.44 (1.000)	1.000	0.997	0.907	0.225
Multi	60	TD3	Static-β	782	-5.93e+00	-11.27 (1.000)	1.000	0.969	0.893	0.683
Window = 90 days										
Equity	90	PPO	Sortino	773	5.54e-02	1.68** (0.046)	0.004***	1.000	0.994	0.987
Equity	90	PPO	Momentum-β	773	-1.08e+00	-11.86 (1.000)	1.000	0.987	0.988	0.869

Continued on next page

Table 1 – continued from previous page

Group	Window	Algo	Comparator	N	Mean Diff (Ann.)	$t_{\text{HAC}}(p)$	Wilcoxon p	t-test p	M-W Up	MBB p_{two}
Equity	90	PPO	Dynamic-β	773	-9.28e-01	-4.83 (1.000)	1.000	0.982	0.978	0.731
Equity	90	PPO	Static-β	773	-8.63e-01	-5.11 (1.000)	1.000	0.986	0.984	0.754
Crypto	90	PPO	Sortino	365	-3.95e+00	-5.94 (1.000)	1.000	0.969	0.957	0.827
Crypto	90	PPO	Momentum-β	365	-4.64e+00	-6.03 (1.000)	1.000	0.946	0.936	0.916
Crypto	90	PPO	Dynamic-β	365	-2.18e+00	-3.58 (1.000)	1.000	0.961	0.956	0.827
Crypto	90	PPO	Static-β	365	2.36e+00	8.81*** (0.000)	0.000***	0.994	0.984	0.911
Macro	90	PPO	Sortino	773	1.31e+00	12.27*** (0.000)	0.000***	0.952	0.974	0.462
Macro	90	PPO	Momentum-β	773	4.77e-01	12.79*** (0.000)	0.000***	0.985	0.993	0.873
Macro	90	PPO	Dynamic-β	773	2.21e+00	13.76*** (0.000)	0.000***	0.928	0.962	0.300
Macro	90	PPO	Static-β	773	2.61e-01	12.70*** (0.000)	0.000***	0.991	0.994	0.916
Multi	90	PPO	Sortino	773	-2.95e+00	-13.76 (1.000)	1.000	0.967	0.967	0.684
Multi	90	PPO	Momentum-β	773	-1.15e+00	-9.53 (1.000)	1.000	0.982	0.971	0.799
Multi	90	PPO	Dynamic-β	773	-1.35e+00	-8.74 (1.000)	1.000	0.979	0.966	0.733
Multi	90	PPO	Static-β	773	-2.58e+00	-14.22 (1.000)	1.000	0.973	0.968	0.728
Multi	90	SAC	Sortino	773	-8.22e+00	-14.64 (1.000)	1.000	0.983	0.983	0.947
Multi	90	SAC	Momentum-β	773	4.01e+01	15.47*** (0.000)	0.000***	0.630	0.600	0.000***
Multi	90	SAC	Dynamic-β	773	1.98e+01	10.93*** (0.000)	0.000***	0.871	0.923	0.013**
Multi	90	SAC	Static-β	773	1.68e+00	2.87*** (0.002)	0.000***	0.941	0.939	0.999
Multi	90	TD3	Sortino	773	-6.24e+00	-4.59 (1.000)	1.000	0.938	0.895	0.011**
Multi	90	TD3	Momentum-β	773	-2.12e+01	-13.33 (1.000)	1.000	0.825	0.820	0.016**
Multi	90	TD3	Dynamic-β	773	4.92e+00	6.30*** (0.000)	0.000***	0.889	0.930	0.074*
Multi	90	TD3	Static-β	773	-5.12e+00	-6.23 (1.000)	1.000	0.942	0.999	0.221
Window = 120 days										
Equity	120	PPO	Sortino	764	-1.16e-01	-2.19 (0.986)	1.000	1.000	0.994	0.937
Equity	120	PPO	Momentum-β	764	-4.00e+00	-12.83 (1.000)	1.000	0.960	0.957	0.616
Equity	120	PPO	Dynamic-β	764	-1.39e+00	-8.53 (1.000)	1.000	0.981	0.978	0.795
Equity	120	PPO	Static-β	764	-1.12e+00	-9.17 (1.000)	1.000	0.985	0.981	0.840
Crypto	120	PPO	Sortino	356	9.43e+00	21.04*** (0.000)	0.000***	0.986	0.986	0.925
Crypto	120	PPO	Momentum-β	356	7.29e+00	19.21*** (0.000)	0.000***	0.998	0.990	0.986
Crypto	120	PPO	Dynamic-β	356	-5.55e+00	-7.73 (1.000)	1.000	0.955	0.972	0.718
Crypto	120	PPO	Static-β	356	7.55e+00	17.77*** (0.000)	0.000***	0.991	0.978	0.884
Macro	120	PPO	Sortino	764	-4.24e-01	-10.68 (1.000)	1.000	0.982	0.987	0.766
Macro	120	PPO	Momentum-β	764	-6.05e-01	-7.86 (1.000)	1.000	0.971	0.968	0.534
Macro	120	PPO	Dynamic-β	764	1.91e+00	12.48*** (0.000)	0.000***	0.932	0.959	0.275
Macro	120	PPO	Static-β	764	1.51e+00	13.27*** (0.000)	0.000***	0.950	0.967	0.410
Multi	120	PPO	Sortino	764	-3.28e+00	-16.33 (1.000)	1.000	0.969	0.971	0.739
Multi	120	PPO	Momentum-β	764	-1.30e+00	-9.90 (1.000)	1.000	0.986	0.980	0.806
Multi	120	PPO	Dynamic-β	764	-3.01e+00	-17.80 (1.000)	1.000	0.973	0.978	0.782
Multi	120	PPO	Static-β	764	-4.89e+00	-15.70 (1.000)	1.000	0.954	0.949	0.587
Multi	120	SAC	Sortino	764	2.02e+01	12.57*** (0.000)	0.000***	0.719	0.828	0.000***
Multi	120	SAC	Momentum-β	764	-2.40e+01	-14.59 (1.000)	1.000	0.698	0.572	0.003***
Multi	120	SAC	Dynamic-β	764	-3.18e+01	-15.22 (1.000)	1.000	0.619	0.522	0.001***
Multi	120	SAC	Static-β	764	1.02e+01	14.02*** (0.000)	0.000***	0.857	0.883	0.136
Multi	120	TD3	Sortino	764	-8.97e+00	-4.35 (1.000)	1.000	0.836	0.852	0.000***
Multi	120	TD3	Momentum-β	764	-9.56e+00	-8.47 (1.000)	1.000	0.813	0.769	0.080*
Multi	120	TD3	Dynamic-β	764	9.54e+00	13.06*** (0.000)	0.000***	0.951	0.883	0.320
Multi	120	TD3	Static-β	764	-3.27e+00	-4.93 (1.000)	1.000	0.955	0.985	0.257

Notes: Mean Diff (Ann.) is the annualized excess return difference between the Sharpe (SR) baseline and the comparator strategy. t_{HAC} uses Newey–West HAC variance. Wilcoxon is a two-sided signed-rank p. t-test p is the p-value from a two-sample t-test. M-W Up is the p-value from a Mann-Whitney U-test. MBB p_{two} is the moving-block bootstrap two-sided p-value. Significance: * $p < 0.10$, ** $p < 0.05$, *** $p < 0.01$.
