# Supplementary material for: Factor-based deep reinforcement learning for asset allocation: Comparative analysis of static and dynamic beta reward designs
Source: PLoS One. 2025 Dec 30;20(12):e0332779. doi: 10.1371/journal.pone.0332779 (PMC12753089; doi:10.1371/journal.pone.0332779)
Supplement: S3 Table — (PDF) [file pone.0332779.s003.pdf]

S3 Table. Full-period performance of all strategies (Equity, Crypto, Macro, Multi-Asset) across window lengths (30, 60, 90, 120 days)

Strategy	Window	Sharpe	Sortino	CumRet	AnnRet	Vol	CAGR	MDD	Turn./Cost
**Panel A: Equity Strategies**									
Equal-Weight	30	0.995	1.659	73.28%	19.50%	19.59%	19.22%	-18.73%	0.010 / 0.25%
	60	1.133	1.915	85.47%	21.84%	19.27%	22.12%	-18.73%	0.010 / 0.25%
	90	1.144	1.947	83.83%	21.73%	19.00%	22.05%	-18.73%	0.010 / 0.24%
	120	1.360	2.364	104.16%	25.38%	18.66%	26.66%	-18.73%	0.010 / 0.24%
Mean-Variance	30	0.379	0.600	13.18%	4.97%	13.14%	4.20%	-16.03%	0.119 / 2.99%
	60	0.298	0.468	9.39%	3.85%	12.94%	3.06%	-16.03%	0.119 / 3.00%
	90	0.298	0.468	9.30%	3.87%	12.98%	3.07%	-16.03%	0.119 / 3.00%
	120	0.285	0.448	8.57%	3.65%	12.85%	2.87%	-16.03%	0.120 / 3.02%
Sharpe-PPO	30	1.241	2.133	91.86%	22.41%	18.06%	23.10%	-18.89%	0.019 / 0.49%
	60	1.280	2.209	92.97%	22.80%	17.81%	23.63%	-18.71%	0.020 / 0.51%
	90	1.296	2.245	92.25%	22.90%	17.67%	23.78%	-18.80%	0.020 / 0.50%
	120	1.361	2.359	95.94%	23.74%	17.44%	24.88%	-18.87%	0.021 / 0.53%
Sortino-PPO	30	1.231	2.117	90.58%	22.20%	18.02%	22.84%	-18.70%	0.019 / 0.49%
	60	1.279	2.206	93.07%	22.82%	17.84%	23.65%	-18.92%	0.020 / 0.52%
	90	1.297	2.247	92.23%	22.89%	17.65%	23.78%	-18.73%	0.020 / 0.51%
	120	1.365	2.365	96.03%	23.74%	17.40%	24.90%	-18.79%	0.021 / 0.53%
Momentum-β	30	1.228	2.108	90.66%	22.22%	18.10%	22.86%	-18.79%	0.020 / 0.49%
	60	1.278	2.205	93.03%	22.82%	17.86%	23.64%	-18.81%	0.020 / 0.50%
	90	1.307	2.266	93.59%	23.13%	17.69%	24.06%	-18.93%	0.020 / 0.50%
	120	1.399	2.430	100.17%	24.45%	17.48%	25.76%	-18.81%	0.022 / 0.55%
Dynamic-β	30	1.224	2.102	89.16%	21.94%	17.92%	22.55%	-18.63%	0.019 / 0.49%
	60	1.279	2.205	92.74%	22.75%	17.80%	23.58%	-18.69%	0.019 / 0.48%
	90	1.317	2.283	94.23%	23.23%	17.64%	24.20%	-18.82%	0.020 / 0.50%
	120	1.381	2.395	97.96%	24.07%	17.43%	25.30%	-18.81%	0.021 / 0.52%
Static-β	30	1.230	2.115	90.05%	22.10%	17.96%	22.73%	-18.73%	0.019 / 0.49%
	60	1.279	2.207	93.13%	22.83%	17.85%	23.66%	-18.76%	0.019 / 0.48%
	90	1.315	2.281	93.83%	23.15%	17.60%	24.11%	-18.72%	0.021 / 0.52%
	120	1.377	2.387	97.55%	24.01%	17.43%	25.21%	-18.85%	0.021 / 0.52%
**Panel B: Crypto-Asset Strategies**									
Equal-Weight	30	1.141	2.027	108.44%	64.80%	56.80%	62.76%	-43.44%	0.013 / 0.34%
	60	0.932	1.648	72.26%	53.12%	56.98%	44.69%	-43.44%	0.013 / 0.33%
	90	0.952	1.686	72.84%	54.32%	57.09%	46.36%	-43.44%	0.013 / 0.33%
	120	0.990	1.760	76.03%	56.68%	57.23%	49.73%	-43.44%	0.013 / 0.33%
Mean-Variance	30	1.121	2.047	88.17%	59.23%	52.83%	57.44%	-33.09%	0.061 / 1.54%
	60	0.954	1.740	64.68%	50.82%	53.28%	44.42%	-33.09%	0.062 / 1.55%
	90	0.649	1.166	31.07%	34.37%	52.97%	22.72%	-33.09%	0.061 / 1.54%
	120	0.394	0.698	9.47%	20.59%	52.32%	7.29%	-33.09%	0.060 / 1.50%
Sharpe-PPO	30	0.872	1.520	60.70%	43.94%	50.38%	36.74%	-41.91%	0.053 / 1.34%
	60	0.816	1.419	52.48%	41.20%	50.47%	32.98%	-41.72%	0.049 / 1.24%
	90	0.794	1.385	48.42%	39.97%	50.36%	31.44%	-42.81%	0.056 / 1.40%
	120	0.801	1.396	48.31%	41.10%	51.32%	32.28%	-42.84%	0.046 / 1.16%
Sortino-PPO	30	0.879	1.535	61.83%	44.59%	50.75%	37.38%	-42.36%	0.051 / 1.29%
	60	0.818	1.421	53.22%	41.92%	51.25%	33.41%	-41.91%	0.043 / 1.09%
	90	0.855	1.498	54.43%	42.25%	49.43%	35.10%	-40.73%	0.056 / 1.42%
	120	0.787	1.368	46.59%	40.03%	50.85%	31.19%	-41.80%	0.049 / 1.24%

Continued on next page

Table 1 – continued from previous page

Strategy	Window	Sharpe	Sortino	CumRet	AnnRet	Vol	CAGR	MDD	Turn./Cost
Momentum-β	30	0.880	1.551	62.46%	45.06%	51.19%	37.73%	-42.45%	0.049 / 1.24%
	60	0.804	1.408	51.88%	41.53%	51.69%	32.62%	-44.72%	0.052 / 1.32%
	90	0.867	1.514	56.80%	43.97%	50.75%	36.53%	-39.31%	0.038 / 0.95%
	120	0.803	1.407	48.58%	41.23%	51.34%	32.45%	-43.03%	0.045 / 1.13%
Dynamic-β	30	0.826	1.434	56.34%	42.95%	51.99%	34.28%	-44.17%	0.046 / 1.15%
	60	0.802	1.391	51.25%	41.00%	51.13%	32.25%	-43.19%	0.051 / 1.29%
	90	0.844	1.475	54.32%	42.89%	50.80%	35.04%	-40.49%	0.042 / 1.06%
	120	0.868	1.527	55.72%	44.54%	51.31%	36.94%	-41.64%	0.043 / 1.09%
Static-β	30	0.851	1.487	58.99%	43.73%	51.36%	35.78%	-42.80%	0.048 / 1.21%
	60	0.807	1.411	51.81%	41.14%	50.97%	32.58%	-43.19%	0.054 / 1.37%
	90	0.774	1.343	46.73%	39.53%	51.06%	30.40%	-43.98%	0.050 / 1.25%
	120	0.799	1.398	48.69%	41.79%	52.32%	32.53%	-42.97%	0.039 / 0.99%
**Panel C: Macro-Asset Strategies**									
Equal-Weight	30	0.585	1.050	21.31%	6.87%	11.75%	6.37%	-13.17%	0.005 / 0.12%
	60	0.678	1.219	25.13%	7.94%	11.71%	7.52%	-13.17%	0.005 / 0.12%
	90	0.661	1.192	24.07%	7.74%	11.72%	7.31%	-12.77%	0.005 / 0.12%
	120	0.751	1.370	27.64%	8.76%	11.66%	8.42%	-12.60%	0.005 / 0.12%
Mean-Variance	30	0.210	0.364	3.61%	1.40%	6.67%	1.18%	-9.30%	0.022 / 0.56%
	60	0.251	0.435	4.41%	1.67%	6.67%	1.46%	-9.30%	0.022 / 0.55%
	90	0.299	0.519	5.30%	1.98%	6.61%	1.77%	-9.30%	0.021 / 0.54%
	120	0.206	0.356	3.38%	1.36%	6.60%	1.15%	-9.30%	0.021 / 0.53%
Sharpe-PPO	30	0.793	1.394	22.72%	6.91%	8.72%	6.75%	-10.48%	0.092 / 2.31%
	60	0.836	1.470	23.15%	7.08%	8.47%	6.95%	-9.98%	0.103 / 2.59%
	90	0.816	1.429	21.85%	6.80%	8.33%	6.66%	-10.02%	0.104 / 2.61%
	120	0.836	1.461	21.96%	6.90%	8.25%	6.78%	-9.92%	0.111 / 2.80%
Sortino-PPO	30	0.808	1.421	23.20%	7.04%	8.71%	6.88%	-10.38%	0.093 / 2.33%
	60	0.827	1.456	22.65%	6.94%	8.39%	6.81%	-9.86%	0.107 / 2.69%
	90	0.769	1.342	20.37%	6.40%	8.32%	6.24%	-10.21%	0.105 / 2.65%
	120	0.847	1.480	22.50%	7.05%	8.32%	6.93%	-9.99%	0.108 / 2.71%
Momentum-β	30	0.747	1.313	20.44%	6.29%	8.41%	6.11%	-10.13%	0.100 / 2.52%
	60	0.772	1.355	20.68%	6.41%	8.30%	6.25%	-10.00%	0.101 / 2.54%
	90	0.792	1.384	21.34%	6.67%	8.42%	6.52%	-10.29%	0.099 / 2.49%
	120	0.838	1.460	22.79%	7.15%	8.53%	7.02%	-10.42%	0.093 / 2.34%
Dynamic-β	30	0.771	1.356	21.66%	6.62%	8.59%	6.45%	-10.30%	0.101 / 2.55%
	60	0.706	1.232	18.78%	5.90%	8.36%	5.71%	-10.39%	0.104 / 2.62%
	90	0.736	1.281	19.58%	6.19%	8.41%	5.99%	-10.49%	0.095 / 2.40%
	120	0.766	1.331	19.85%	6.32%	8.26%	5.98%	-10.21%	0.108 / 2.72%
Static-β	30	0.774	1.361	21.34%	6.53%	8.44%	6.37%	-10.05%	0.098 / 2.47%
	60	0.747	1.310	19.81%	6.17%	8.26%	6.01%	-10.01%	0.102 / 2.56%
	90	0.817	1.432	21.61%	6.73%	8.23%	6.60%	-9.88%	0.103 / 2.61%
	120	0.789	1.375	20.42%	6.47%	8.21%	6.33%	-10.07%	0.100 / 2.52%
**Panel D: Multi-Asset Strategies (PPO-based)**									
Equal-Weight	30	1.072	1.777	68.75%	18.17%	16.95%	18.21%	-16.70%	0.010 / 0.25%
	60	1.231	2.076	80.57%	20.51%	16.66%	21.07%	-16.70%	0.010 / 0.25%
	90	1.234	2.096	78.62%	20.34%	16.48%	20.91%	-16.70%	0.010 / 0.25%
	120	1.475	2.576	97.16%	23.78%	16.12%	25.21%	-16.70%	0.010 / 0.25%
Mean-Variance	30	0.378	0.631	7.49%	2.64%	6.99%	2.43%	-10.46%	0.082 / 2.07%
	60	0.360	0.599	6.99%	2.51%	6.99%	2.30%	-10.46%	0.082 / 2.07%

Continued on next page

Table 1 – continued from previous page

Strategy	Window	Sharpe	Sortino	CumRet	AnnRet	Vol	CAGR	MDD	Turn./Cost
	90	0.368	0.610	7.04%	2.56%	6.96%	2.34%	-10.46%	0.082 / 2.08%
	120	0.308	0.510	5.63%	2.12%	6.89%	1.90%	-10.46%	0.082 / 2.07%
Sharpe-PPO	30	1.352	2.329	88.06%	21.40%	15.83%	22.32%	-15.92%	0.016 / 0.41%
	60	1.409	2.441	89.75%	21.88%	15.52%	22.96%	-15.79%	0.017 / 0.42%
	90	1.436	2.503	89.36%	22.02%	15.34%	23.17%	-15.68%	0.016 / 0.41%
	120	1.487	2.594	91.79%	22.67%	15.25%	24.00%	-15.87%	0.017 / 0.42%
Sortino-PPO	30	1.299	2.226	82.32%	20.39%	15.70%	21.12%	-15.90%	0.017 / 0.43%
	60	1.409	2.440	89.90%	21.90%	15.54%	22.99%	-15.90%	0.017 / 0.42%
	90	1.457	2.545	92.28%	22.54%	15.47%	23.79%	-15.96%	0.017 / 0.42%
	120	1.521	2.669	94.59%	23.15%	15.22%	24.59%	-15.81%	0.017 / 0.43%
Momentum-β	30	1.352	2.328	88.43%	21.47%	15.88%	22.40%	-15.91%	0.016 / 0.41%
	60	1.409	2.433	90.94%	22.10%	15.68%	23.21%	-15.87%	0.016 / 0.41%
	90	1.432	2.489	90.77%	22.30%	15.57%	23.47%	-15.88%	0.016 / 0.39%
	120	1.483	2.588	92.92%	22.90%	15.44%	24.24%	-16.06%	0.016 / 0.40%
Dynamic-β	30	1.337	2.302	87.16%	21.26%	15.90%	22.13%	-16.04%	0.016 / 0.41%
	60	1.397	2.416	89.02%	21.76%	15.57%	22.81%	-15.89%	0.016 / 0.41%
	90	1.442	2.509	91.16%	22.35%	15.50%	23.55%	-15.86%	0.016 / 0.41%
	120	1.513	2.648	94.23%	23.09%	15.27%	24.51%	-15.80%	0.016 / 0.42%
Static-β	30	1.342	2.309	87.48%	21.31%	15.88%	22.20%	-16.01%	0.017 / 0.43%
	60	1.371	2.367	86.32%	21.29%	15.52%	22.24%	-15.91%	0.018 / 0.44%
	90	1.449	2.531	91.71%	22.45%	15.49%	23.67%	-15.96%	0.017 / 0.42%
	120	1.527	2.681	95.91%	23.39%	15.31%	24.87%	-15.89%	0.017 / 0.44%
**Panel E: Multi-Asset Strategies (SAC-based)**									
Sharpe-SAC	30	1.075	1.841	62.69%	16.74%	15.57%	16.79%	-14.89%	0.018 / 0.45%
	60	1.274	2.212	81.46%	20.53%	16.11%	21.20%	-17.50%	0.026 / 0.67%
	90	1.713	3.058	109.64%	25.25%	14.74%	27.33%	-14.86%	0.029 / 0.73%
	120	1.448	2.521	73.64%	19.10%	13.19%	19.99%	-12.10%	0.023 / 0.57%
Sortino-SAC	30	1.185	2.041	82.70%	20.76%	17.52%	21.20%	-18.23%	0.019 / 0.48%
	60	1.326	2.284	78.98%	19.91%	15.02%	20.66%	-14.94%	0.018 / 0.46%
	90	1.609	2.868	110.25%	25.52%	15.86%	27.45%	-17.01%	0.020 / 0.51%
	120	1.162	1.992	54.62%	15.25%	13.13%	15.48%	-15.00%	0.029 / 0.72%
Momentum-β	30	1.221	2.103	74.56%	18.98%	15.54%	19.45%	-15.80%	0.016 / 0.41%
	60	1.222	2.121	77.03%	19.73%	16.15%	20.24%	-16.58%	0.018 / 0.46%
	90	1.383	2.420	76.86%	19.62%	14.18%	20.46%	-14.34%	0.023 / 0.59%
	120	1.585	2.788	97.17%	23.53%	14.84%	25.13%	-15.23%	0.021 / 0.54%
Dynamic-β	30	1.199	2.079	79.95%	20.15%	16.81%	20.61%	-17.05%	0.019 / 0.48%
	60	1.314	2.284	73.86%	18.88%	14.37%	19.54%	-14.74%	0.021 / 0.53%
	90	1.546	2.721	97.17%	23.30%	15.08%	24.81%	-15.01%	0.019 / 0.49%
	120	1.609	2.839	105.00%	24.91%	15.48%	26.76%	-16.48%	0.020 / 0.50%
Static-β	30	1.231	2.114	64.85%	16.89%	13.72%	17.29%	-14.24%	0.014 / 0.36%
	60	1.321	2.314	86.92%	21.51%	16.28%	22.36%	-16.90%	0.024 / 0.59%
	90	1.583	2.828	113.89%	26.19%	16.55%	28.17%	-16.61%	0.039 / 0.98%
	120	1.296	2.263	63.73%	17.16%	13.24%	17.68%	-14.62%	0.020 / 0.51%
**Panel F: Multi-Asset Strategies (TD3-based)**									
Sharpe-TD3	30	1.326	2.305	93.07%	22.42%	16.90%	23.35%	-18.59%	0.029 / 0.73%
	60	1.516	2.647	91.66%	22.05%	14.55%	23.36%	-14.90%	0.026 / 0.65%
	90	1.238	2.162	82.89%	21.17%	17.10%	21.78%	-17.67%	0.031 / 0.77%
	120	1.334	2.304	89.00%	22.44%	16.83%	23.40%	-17.55%	0.026 / 0.66%

Continued on next page

Table 1 – continued from previous page

**Strategy**	**Window**	**Sharpe**	**Sortino**	**CumRet**	**AnnRet**	**Vol**	**CAGR**	**MDD**	**Turn./Cost**
Sortino-TD3	30	1.341	2.345	68.41%	17.48%	13.04%	18.09%	-12.73%	0.028 / 0.70%
	60	1.588	2.785	87.74%	21.22%	13.36%	22.54%	-13.99%	0.032 / 0.81%
	90	1.509	2.637	90.79%	22.17%	14.69%	23.47%	-15.44%	0.021 / 0.53%
	120	1.614	2.864	106.53%	25.17%	15.59%	27.07%	-16.38%	0.026 / 0.65%
Momentum-β	30	1.203	2.095	74.27%	18.96%	15.76%	19.38%	-16.14%	0.028 / 0.71%
	60	1.272	2.215	71.12%	18.38%	14.44%	18.93%	-14.80%	0.029 / 0.73%
	90	1.449	2.520	100.97%	24.18%	16.69%	25.59%	-17.49%	0.034 / 0.86%
	120	1.525	2.636	108.37%	25.67%	16.84%	27.44%	-18.49%	0.028 / 0.70%
Dynamic-β	30	1.322	2.299	89.21%	21.69%	16.40%	22.56%	-17.98%	0.027 / 0.69%
	60	1.545	2.749	92.12%	22.09%	14.30%	22.95%	-14.39%	0.026 / 0.66%
	90	1.175	2.031	73.20%	19.27%	16.40%	19.64%	-17.44%	0.026 / 0.64%
	120	1.439	2.504	86.11%	21.65%	15.05%	22.77%	-15.75%	0.025 / 0.63%
Static-β	30	1.054	1.817	64.78%	17.27%	13.72%	17.27%	-14.24%	0.026 / 0.66%
	60	1.461	2.581	88.78%	21.60%	14.78%	21.59%	-14.98%	0.028 / 0.71%
	90	1.375	2.432	89.35%	22.14%	16.10%	23.17%	-16.67%	0.030 / 0.76%
	120	1.456	2.544	94.21%	23.19%	15.93%	24.51%	-16.29%	0.018 / 0.45%
