# Supplementary material for: Factor-based deep reinforcement learning for asset allocation: Comparative analysis of static and dynamic beta reward designs
Source: PLoS One. 2025 Dec 30;20(12):e0332779. doi: 10.1371/journal.pone.0332779 (PMC12753089; doi:10.1371/journal.pone.0332779)
Supplement: S4 Table — (PDF) [file pone.0332779.s004.pdf]

S4 Table. Turnover and realized transaction costs across domains, algorithms, and window lengths

**Domain**	**Algorithm**	**Window** (days)	**Mean Turn.** (daily, %)	**Annual cost** (%/year)	**Daily cost** (%/day)	**Cost** (bps)	**Gross** Sharpe	**Net** Sharpe	**Gross** AnnRet (%)	**Net** AnnRet (%)	**Net−Gross** (pp)	**Gross Vol** (%/year)
equity	EqualWeight	30	0.98%	24.59%	0.098%	10	1.038	-0.245	20.40%	-4.80%	-25.20%	19.64%
equity	EqualWeight	60	0.97%	24.52%	0.097%	10	1.161	-0.145	22.40%	-2.80%	-25.20%	19.29%
equity	EqualWeight	90	0.97%	24.48%	0.097%	10	1.140	-0.187	21.64%	-3.56%	-25.20%	18.98%
equity	EqualWeight	120	0.97%	24.47%	0.097%	10	1.272	-0.067	23.93%	-1.27%	-25.20%	18.82%
equity	MeanVariance	30	11.86%	298.91%	1.186%	10	0.379	-1.541	4.98%	-20.22%	-25.20%	13.13%
equity	MeanVariance	60	11.91%	300.12%	1.191%	10	0.291	-1.657	3.76%	-21.44%	-25.20%	12.94%
equity	MeanVariance	90	11.90%	299.94%	1.190%	10	0.308	-1.634	4.00%	-21.20%	-25.20%	12.97%
equity	MeanVariance	120	11.97%	301.68%	1.197%	10	0.331	-1.625	4.27%	-20.93%	-25.20%	12.88%
equity	PPO	30	1.95%	49.04%	0.195%	10	1.230	-0.170	22.15%	-3.05%	-25.20%	18.00%
equity	PPO	60	1.97%	49.66%	0.197%	10	1.277	-0.137	22.76%	-2.44%	-25.20%	17.82%
equity	PPO	90	2.00%	50.50%	0.200%	10	1.307	-0.121	23.06%	-2.14%	-25.20%	17.64%
equity	PPO	120	2.10%	52.84%	0.210%	10	1.373	-0.073	23.93%	-1.27%	-25.20%	17.42%
crypto	EqualWeight	30	0.00%	0.00%	0.000%	10	1.171	-0.340	65.10%	39.90%	-25.20%	56.80%
crypto	EqualWeight	60	0.00%	0.00%	0.000%	10	0.960	-0.389	53.40%	28.20%	-25.20%	57.03%
crypto	EqualWeight	90	0.00%	0.00%	0.000%	10	0.926	-0.453	52.10%	26.90%	-25.20%	57.15%
crypto	EqualWeight	120	0.00%	0.00%	0.000%	10	0.920	-0.471	53.17%	27.97%	-25.20%	57.29%
crypto	MeanVariance	30	6.05%	152.44%	0.605%	10	1.136	-0.682	58.52%	33.32%	-25.20%	52.80%
crypto	MeanVariance	60	6.02%	151.73%	0.602%	10	0.968	-0.865	50.66%	25.46%	-25.20%	53.26%
crypto	MeanVariance	90	6.04%	152.23%	0.604%	10	0.660	-1.210	34.17%	8.97%	-25.20%	52.97%
crypto	MeanVariance	120	5.98%	150.77%	0.598%	10	0.412	-1.453	20.59%	-4.61%	-25.20%	52.32%
crypto	PPO	30	4.96%	124.90%	0.496%	10	0.864	-0.778	43.07%	17.87%	-25.20%	50.40%
crypto	PPO	60	4.89%	122.37%	0.489%	10	0.820	-0.831	40.52%	15.32%	-25.20%	50.45%
crypto	PPO	90	5.69%	142.19%	0.569%	10	0.752	-0.901	38.18%	12.98%	-25.20%	50.33%
crypto	PPO	120	4.69%	117.17%	0.469%	10	0.764	-0.804	40.75%	15.55%	-25.20%	51.22%
macro	EqualWeight	30	0.47%	11.74%	0.047%	10	0.583	-1.937	6.88%	-18.32%	-25.20%	11.76%
macro	EqualWeight	60	0.47%	11.73%	0.047%	10	0.674	-1.830	7.95%	-17.25%	-25.20%	11.72%
macro	EqualWeight	90	0.47%	11.75%	0.047%	10	0.651	-1.848	7.74%	-17.46%	-25.20%	11.73%
macro	EqualWeight	120	0.47%	11.75%	0.047%	10	0.762	-1.740	8.79%	-16.41%	-25.20%	11.68%
macro	MeanVariance	30	2.90%	72.41%	0.290%	10	0.206	-3.974	1.43%	-23.77%	-25.20%	6.68%
macro	MeanVariance	60	2.90%	72.53%	0.290%	10	0.254	-3.971	1.68%	-23.52%	-25.20%	6.69%
macro	MeanVariance	90	2.91%	72.73%	0.291%	10	0.306	-3.956	1.97%	-23.23%	-25.20%	6.67%
macro	MeanVariance	120	2.89%	72.30%	0.289%	10	0.223	-4.055	1.43%	-23.77%	-25.20%	6.67%
macro	PPO	30	9.23%	230.71%	0.923%	10	0.793	-2.727	6.90%	-18.30%	-25.20%	8.72%
macro	PPO	60	10.32%	257.98%	1.032%	10	0.835	-2.744	7.08%	-18.12%	-25.20%	8.47%
macro	PPO	90	10.38%	259.51%	1.038%	10	0.817	-2.768	6.81%	-18.39%	-25.20%	8.38%
macro	PPO	120	10.98%	274.58%	1.098%	10	0.842	-2.729	6.94%	-18.26%	-25.20%	8.26%
multi	EqualWeight	30	0.99%	24.91%	0.099%	10	1.117	-0.365	18.99%	-6.21%	-25.20%	17.00%
multi	EqualWeight	60	0.98%	24.82%	0.098%	10	1.256	-0.257	20.93%	-4.27%	-25.20%	16.66%
multi	EqualWeight	90	0.98%	24.74%	0.098%	10	1.233	-0.296	20.32%	-4.88%	-25.20%	16.47%
multi	EqualWeight	120	0.98%	24.70%	0.098%	10	1.360	-0.182	22.23%	-2.97%	-25.20%	16.34%
multi	MeanVariance	30	8.23%	207.44%	0.823%	10	0.384	-3.224	2.68%	-22.52%	-25.20%	6.99%
multi	MeanVariance	60	8.23%	207.33%	0.823%	10	0.351	-3.258	2.45%	-22.75%	-25.20%	6.98%
multi	MeanVariance	90	8.24%	207.64%	0.824%	10	0.360	-3.265	2.50%	-22.70%	-25.20%	6.95%
multi	MeanVariance	120	8.22%	207.24%	0.822%	10	0.356	-3.289	2.46%	-22.74%	-25.20%	6.91%

**Domain**	**Algorithm**	**Window** (days)	**Mean Turn.** (daily, %)	**Annual cost** (%/year)	**Daily cost** (%/day)	**Cost** (bps)	**Gross** Sharpe	**Net** Sharpe	**Gross** AnnRet (%)	**Net** AnnRet (%)	**Net−Gross** (pp)	**Gross Vol** (%/year)
multi	PPO	30	1.66%	41.77%	0.166%	10	1.336	-0.257	21.14%	-4.06%	-25.20%	15.83%
multi	PPO	60	1.66%	41.88%	0.166%	10	1.399	-0.221	21.77%	-3.43%	-25.20%	15.56%
multi	PPO	90	1.63%	41.20%	0.163%	10	1.443	-0.187	22.31%	-2.89%	-25.20%	15.46%
multi	PPO	120	1.68%	42.24%	0.168%	10	1.500	-0.148	22.94%	-2.26%	-25.20%	15.29%
multi	SAC	30	1.72%	43.44%	0.172%	10	1.180	-0.424	18.65%	-6.55%	-25.20%	15.82%
multi	SAC	60	2.15%	54.30%	0.215%	10	1.292	-0.330	20.10%	-5.10%	-25.20%	15.58%
multi	SAC	90	2.61%	65.69%	0.261%	10	1.566	-0.089	23.95%	-1.25%	-25.20%	15.27%
multi	SAC	120	2.26%	56.89%	0.226%	10	1.414	-0.399	19.90%	-5.30%	-25.20%	13.97%
multi	TD3	30	2.78%	69.95%	0.278%	10	1.248	-0.372	19.53%	-5.67%	-25.20%	15.69%
multi	TD3	60	2.83%	71.31%	0.283%	10	1.475	-0.292	21.03%	-4.17%	-25.20%	14.28%
multi	TD3	90	2.83%	71.28%	0.283%	10	1.350	-0.211	21.78%	-3.42%	-25.20%	16.18%
multi	TD3	120	2.45%	61.73%	0.245%	10	1.469	-0.106	23.54%	-1.66%	-25.20%	16.04%
