# Supplementary material for: Factor-based deep reinforcement learning for asset allocation: Comparative analysis of static and dynamic beta reward designs
Source: PLoS One. 2025 Dec 30;20(12):e0332779. doi: 10.1371/journal.pone.0332779 (PMC12753089; doi:10.1371/journal.pone.0332779)
Supplement: S5 Table — (PDF) [file pone.0332779.s005.pdf]

S5 Table. Beta-window sensitivity of DRL strategies (Sharpe ratio by beta estimation window)

Asset Group	Reward (Algo)	30-day	60-day	90-day	120-day
**Crypto**					
	Sharpe (PPO)	0.189	0.139	0.096	0.142
	Sortino (PPO)	0.181	0.149	0.146	0.132
	Momentum-β (PPO)	0.103	0.104	0.090	0.067
	Dynamic-β (PPO)	0.130	0.091	0.085	0.092
	Static-β (PPO)	0.084	0.104	0.117	0.089
**Equity**					
	Sharpe (PPO)	1.267	1.309	1.331	1.394
	Sortino (PPO)	1.260	1.311	1.343	1.402
	Momentum-β (PPO)	1.255	1.306	1.339	1.425
	Dynamic-β (PPO)	1.250	1.304	1.342	1.399
	Static-β (PPO)	1.258	1.314	1.344	1.404
**Macro**					
	Sharpe (PPO)	-2.626	-2.652	-2.682	-2.712
	Sortino (PPO)	-2.581	-2.619	-2.710	-2.690
	Momentum-β (PPO)	-2.102	-2.106	-2.032	-1.929
	Dynamic-β (PPO)	-2.005	-2.180	-2.118	-2.127
	Static-β (PPO)	-2.055	-2.155	-2.065	-2.114
**Multi-Asset**					
	Sharpe (PPO)	0.136	0.179	0.190	0.235
	Sortino (PPO)	0.050	0.180	0.231	0.276
	Momentum-β (PPO)	0.141	0.195	0.208	0.248
	Dynamic-β (PPO)	0.122	0.167	0.215	0.270
	Static-β (PPO)	0.127	0.127	0.222	0.293
	Sharpe (SAC)	0.101	0.120	0.159	0.192
	Sortino (SAC)	0.043	0.138	0.202	0.241
	Momentum-β (SAC)	0.110	0.166	0.195	0.232
	Dynamic-β (SAC)	0.097	0.141	0.209	0.262
	Static-β (SAC)	0.102	0.118	0.224	0.278
	Sharpe (TD3)	0.089	0.143	0.176	0.210
	Sortino (TD3)	0.050	0.160	0.218	0.255
	Momentum-β (TD3)	0.098	0.178	0.202	0.238
	Dynamic-β (TD3)	0.086	0.152	0.217	0.261
	Static-β (TD3)	0.091	0.139	0.235	0.281
