# Supplementary material for: Factor-based deep reinforcement learning for asset allocation: Comparative analysis of static and dynamic beta reward designs
Source: PLoS One. 2025 Dec 30;20(12):e0332779. doi: 10.1371/journal.pone.0332779 (PMC12753089; doi:10.1371/journal.pone.0332779)
Supplement: S6 Table — (PDF) [file pone.0332779.s006.pdf]

S6 Table. Regime-conditional performance of PPO-based strategies across equity, crypto, macro, and multi-asset portfolios (volatility regimes, β-window = 60 days)

Domain	Strategy	Regime	Net Sharpe	Net AnnRet (%)	Net Vol (%)	Net CAGR (%)	Net MDD (%)
Equity	Sharpe	HighVol	-0.712	-21.62%	19.49%	-19.14%	-33.39%
	Sharpe	LowVol	1.187	25.68%	18.89%	24.59%	-10.61%
	Sharpe	MidVol	0.257	1.35%	19.06%	1.07%	-18.09%
	Sortino	HighVol	-0.714	-21.64%	19.51%	-19.15%	-33.43%
	Sortino	LowVol	1.187	25.68%	18.89%	24.60%	-10.64%
	Sortino	MidVol	0.267	1.44%	19.06%	1.15%	-18.11%
	Momentum-β	HighVol	-0.709	-21.55%	19.47%	-19.08%	-33.36%
	Momentum-β	LowVol	1.188	25.69%	18.88%	24.60%	-10.61%
	Momentum-β	MidVol	0.264	1.41%	19.05%	1.13%	-18.10%
	Dynamic-β	HighVol	-0.694	-21.16%	19.33%	-18.74%	-33.15%
	Dynamic-β	LowVol	1.185	25.65%	18.88%	24.59%	-10.59%
	Dynamic-β	MidVol	0.246	1.27%	18.98%	0.99%	-18.04%
	Static-β	HighVol	-0.706	-21.52%	19.44%	-19.07%	-33.30%
	Static-β	LowVol	1.185	25.64%	18.88%	24.58%	-10.60%
	Static-β	MidVol	0.259	1.38%	19.05%	1.10%	-18.07%
Crypto	Sharpe	HighVol	-0.801	-19.35%	50.98%	-37.99%	-73.18%
	Sharpe	LowVol	0.664	41.80%	52.32%	40.79%	-27.64%
	Sharpe	MidVol	-0.363	-11.28%	57.53%	-11.76%	-59.79%
	Sortino	HighVol	-0.788	-18.97%	50.93%	-37.64%	-72.82%
	Sortino	LowVol	0.665	41.81%	52.34%	40.80%	-27.62%
	Sortino	MidVol	-0.359	-11.14%	57.50%	-11.62%	-59.74%
	Momentum-β	HighVol	-0.800	-19.30%	50.96%	-37.95%	-73.14%
	Momentum-β	LowVol	0.664	41.80%	52.32%	40.79%	-27.63%
	Momentum-β	MidVol	-0.361	-11.21%	57.51%	-11.69%	-59.76%
	Dynamic-β	HighVol	-0.791	-19.06%	50.93%	-37.75%	-72.94%
	Dynamic-β	LowVol	0.662	41.73%	52.30%	40.73%	-27.60%
	Dynamic-β	MidVol	-0.356	-11.09%	57.53%	-11.59%	-59.73%
	Static-β	HighVol	-0.805	-19.44%	50.99%	-38.06%	-73.20%
	Static-β	LowVol	0.665	41.83%	52.34%	40.82%	-27.65%
	Static-β	MidVol	-0.365	-11.32%	57.51%	-11.80%	-59.80%
Macro	Sharpe	HighVol	-0.200	-1.58%	10.40%	-1.74%	-15.91%
	Sharpe	LowVol	0.930	10.14%	8.82%	10.14%	-4.73%
	Sharpe	MidVol	0.535	3.52%	7.60%	3.50%	-8.61%
	Sortino	HighVol	-0.214	-1.68%	10.41%	-1.80%	-15.93%
	Sortino	LowVol	0.924	10.12%	8.83%	10.12%	-4.72%
	Sortino	MidVol	0.539	3.57%	7.61%	3.55%	-8.63%
	Momentum-β	HighVol	-0.165	-1.30%	10.42%	-1.44%	-15.89%
	Momentum-β	LowVol	0.919	10.08%	8.83%	10.07%	-4.71%
	Momentum-β	MidVol	0.524	3.42%	7.60%	3.40%	-8.59%
	Dynamic-β	HighVol	-0.187	-1.49%	10.40%	-1.61%	-15.89%
	Dynamic-β	LowVol	0.916	10.05%	8.83%	10.05%	-4.71%
	Dynamic-β	MidVol	0.531	3.49%	7.61%	3.47%	-8.60%
	Static-β	HighVol	-0.181	-1.44%	10.40%	-1.57%	-15.90%
	Static-β	LowVol	0.921	10.10%	8.82%	10.09%	-4.72%
	Static-β	MidVol	0.535	3.54%	7.61%	3.52%	-8.61%
Multi-asset	Sharpe	HighVol	-2.757	-74.50%	27.02%	-54.27%	-64.16%
	Sharpe	LowVol	1.330	16.64%	12.51%	17.19%	-4.41%
	Sharpe	MidVol	0.445	7.58%	17.04%	6.32%	-25.95%
	Sortino	HighVol	-2.761	-74.84%	27.11%	-54.49%	-64.27%
	Sortino	LowVol	1.357	17.04%	12.56%	17.65%	-4.34%
	Sortino	MidVol	0.454	7.78%	17.15%	6.52%	-26.13%
	Momentum-β	HighVol	-2.720	-74.38%	27.35%	-54.25%	-64.31%
	Momentum-β	LowVol	1.422	17.88%	12.58%	18.63%	-4.51%
	Momentum-β	MidVol	0.444	7.61%	17.16%	6.34%	-26.33%
	Dynamic-β	HighVol	-2.762	-74.73%	27.05%	-54.38%	-64.21%
	Dynamic-β	LowVol	1.289	16.03%	12.43%	16.48%	-4.43%
	Dynamic-β	MidVol	0.448	7.61%	16.99%	6.37%	-26.12%
	Static-β	HighVol	-2.821	-75.35%	26.71%	-54.62%	-64.42%
	Static-β	LowVol	1.132	13.92%	12.30%	14.07%	-4.57%
	Static-β	MidVol	0.439	7.39%	16.83%	6.16%	-25.69%
