# Supplementary material for: Factor-based deep reinforcement learning for asset allocation: Comparative analysis of static and dynamic beta reward designs
Source: PLoS One. 2025 Dec 30;20(12):e0332779. doi: 10.1371/journal.pone.0332779 (PMC12753089; doi:10.1371/journal.pone.0332779)
Supplement: S7 Table — (PDF) [file pone.0332779.s007.pdf]

S7 Table. Raw pairwise p-values comparing alternative rewards against the Sharpe baseline across asset groups and algorithms.

Domain	Algorithm	Window (days)	Comparator (vs. Sharpe)	HAC t p-value p_{t}	Wilcoxon p-value p_{W}	MBB p-value p_{MBB}
Crypto	PPO	30	Dynamic-β	0.680	0.488	0.511
Crypto	PPO	30	Momentum-β	0.286	0.483	0.518
Crypto	PPO	30	Sortino	0.261	0.494	0.495
Crypto	PPO	30	Static-β	0.541	0.486	0.526
Crypto	PPO	60	Dynamic-β	0.556	0.493	0.498
Crypto	PPO	60	Momentum-β	0.462	0.472	0.508
Crypto	PPO	60	Sortino	0.287	0.505	0.495
Crypto	PPO	60	Static-β	0.514	0.496	0.502
Crypto	PPO	90	Dynamic-β	0.581	0.492	0.512
Crypto	PPO	90	Momentum-β	0.473	0.481	0.517
Crypto	PPO	90	Sortino	0.417	0.500	0.499
Crypto	PPO	90	Static-β	0.605	0.504	0.493
Crypto	PPO	120	Dynamic-β	0.535	0.488	0.514
Crypto	PPO	120	Momentum-β	0.481	0.473	0.527
Crypto	PPO	120	Sortino	0.409	0.486	0.503
Crypto	PPO	120	Static-β	0.589	0.493	0.504
Equity	PPO	30	Dynamic-β	0.654	0.475	0.503
Equity	PPO	30	Momentum-β	0.681	0.493	0.500
Equity	PPO	30	Sortino	0.862	0.482	0.502
Equity	PPO	30	Static-β	0.750	0.493	0.497
Equity	PPO	60	Dynamic-β	0.668	0.492	0.495
Equity	PPO	60	Momentum-β	0.684	0.485	0.507
Equity	PPO	60	Sortino	0.888	0.506	0.492
Equity	PPO	60	Static-β	0.771	0.494	0.505
Equity	PPO	90	Dynamic-β	0.674	0.487	0.502
Equity	PPO	90	Momentum-β	0.696	0.489	0.500
Equity	PPO	90	Sortino	0.900	0.509	0.496
Equity	PPO	90	Static-β	0.782	0.494	0.503
Equity	PPO	120	Dynamic-β	0.689	0.495	0.493
Equity	PPO	120	Momentum-β	0.710	0.486	0.506
Equity	PPO	120	Sortino	0.912	0.504	0.495
Equity	PPO	120	Static-β	0.795	0.492	0.505
Macro	PPO	30	Dynamic-β	0.132	0.482	0.505
Macro	PPO	30	Momentum-β	0.332	0.476	0.511
Macro	PPO	30	Sortino	0.269	0.493	0.499
Macro	PPO	30	Static-β	0.405	0.485	0.506
Macro	PPO	60	Dynamic-β	0.095	0.492	0.501
Macro	PPO	60	Momentum-β	0.288	0.480	0.509
Macro	PPO	60	Sortino	0.239	0.504	0.495
Macro	PPO	60	Static-β	0.380	0.489	0.503
Macro	PPO	90	Dynamic-β	0.088	0.487	0.503
Macro	PPO	90	Momentum-β	0.278	0.479	0.513
Macro	PPO	90	Sortino	0.228	0.498	0.498
Macro	PPO	90	Static-β	0.372	0.487	0.506

Continued on next page

**Domain**	**Algorithm**	**Window** (days)	**Comparator** (vs. Sharpe)	**HAC t p-value** p_{t}	**Wilcoxon p-value** p_{W}	**MBB p-value** p_{MBB}
Macro	PPO	120	Dynamic-β	0.081	0.492	0.504
Macro	PPO	120	Momentum-β	0.270	0.481	0.512
Macro	PPO	120	Sortino	0.221	0.501	0.498
Macro	PPO	120	Static-β	0.365	0.489	0.508
Multi	PPO	30	Dynamic-β	0.192	0.493	0.497
Multi	PPO	30	Momentum-β	0.267	0.480	0.507
Multi	PPO	30	Sortino	0.257	0.496	0.494
Multi	PPO	30	Static-β	0.316	0.487	0.503
Multi	PPO	60	Dynamic-β	0.167	0.492	0.498
Multi	PPO	60	Momentum-β	0.254	0.478	0.509
Multi	PPO	60	Sortino	0.244	0.502	0.495
Multi	PPO	60	Static-β	0.303	0.486	0.503
Multi	PPO	90	Dynamic-β	0.158	0.491	0.502
Multi	PPO	90	Momentum-β	0.246	0.477	0.512
Multi	PPO	90	Sortino	0.235	0.501	0.496
Multi	PPO	90	Static-β	0.295	0.485	0.506
Multi	PPO	120	Dynamic-β	0.148	0.493	0.501
Multi	PPO	120	Momentum-β	0.238	0.479	0.511
Multi	PPO	120	Sortino	0.228	0.503	0.497
Multi	PPO	120	Static-β	0.289	0.487	0.506
Multi	SAC	30	Dynamic-β	0.214	0.486	0.502
Multi	SAC	30	Momentum-β	0.345	0.478	0.511
Multi	SAC	30	Sortino	0.296	0.496	0.495
Multi	SAC	30	Static-β	0.389	0.487	0.504
Multi	SAC	60	Dynamic-β	0.187	0.490	0.500
Multi	SAC	60	Momentum-β	0.321	0.479	0.509
Multi	SAC	60	Sortino	0.273	0.503	0.494
Multi	SAC	60	Static-β	0.373	0.486	0.504
Multi	SAC	90	Dynamic-β	0.176	0.491	0.502
Multi	SAC	90	Momentum-β	0.311	0.480	0.510
Multi	SAC	90	Sortino	0.265	0.504	0.495
Multi	SAC	90	Static-β	0.362	0.487	0.505
Multi	SAC	120	Dynamic-β	0.163	0.492	0.503
Multi	SAC	120	Momentum-β	0.301	0.481	0.511
Multi	SAC	120	Sortino	0.254	0.505	0.497
Multi	SAC	120	Static-β	0.351	0.488	0.506
Multi	TD3	30	Dynamic-β	0.226	0.488	0.502
Multi	TD3	30	Momentum-β	0.357	0.479	0.511
Multi	TD3	30	Sortino	0.309	0.498	0.495
Multi	TD3	30	Static-β	0.402	0.489	0.504
Multi	TD3	60	Dynamic-β	0.199	0.491	0.500
Multi	TD3	60	Momentum-β	0.334	0.480	0.509
Multi	TD3	60	Sortino	0.286	0.504	0.494
Multi	TD3	60	Static-β	0.383	0.487	0.505
Multi	TD3	90	Dynamic-β	0.187	0.492	0.501
Multi	TD3	90	Momentum-β	0.323	0.481	0.510
Multi	TD3	90	Sortino	0.275	0.505	0.495

Continued on next page

**Domain**	**Algorithm**	**Window** (days)	**Comparator** (vs. Sharpe)	**HAC t p-value** p_{t}	**Wilcoxon p-value** p_{W}	**MBB p-value** p_{MBB}
Multi	TD3	90	Static-β	0.372	0.488	0.505
Multi	TD3	120	Dynamic-β	0.176	0.493	0.503
Multi	TD3	120	Momentum-β	0.312	0.482	0.511
Multi	TD3	120	Sortino	0.264	0.506	0.497
Multi	TD3	120	Static-β	0.361	0.489	0.506
