# Supplementary material for: Factor-based deep reinforcement learning for asset allocation: Comparative analysis of static and dynamic beta reward designs
Source: PLoS One. 2025 Dec 30;20(12):e0332779. doi: 10.1371/journal.pone.0332779 (PMC12753089; doi:10.1371/journal.pone.0332779)
Supplement: S8 Table — (PDF) [file pone.0332779.s008.pdf]

S8 Table. Pairwise comparisons including Sharpe differences and three statistical tests (HAC t, Wilcoxon, MBB).

Domain	Algorithm	Window	Comparator	ΔSharpe	p_t	p_W	p_{boot}
equity	PPO	30	Sortino	-0.000	0.980	0.998	0.513
equity	PPO	30	Momentum-β	-0.000	0.979	0.998	0.513
equity	PPO	30	Dynamic-β	0.000	0.979	0.998	0.513
equity	PPO	30	Static-β	-0.000	0.979	0.998	0.513
equity	PPO	60	Sortino	0.000	0.984	0.998	0.510
equity	PPO	60	Momentum-β	0.000	0.984	0.998	0.510
equity	PPO	60	Dynamic-β	-0.000	0.984	0.998	0.510
equity	PPO	60	Static-β	-0.000	0.984	0.998	0.510
equity	PPO	90	Sortino	-0.000	0.982	0.998	0.507
equity	PPO	90	Momentum-β	0.000	0.982	0.998	0.507
equity	PPO	90	Dynamic-β	0.000	0.982	0.998	0.507
equity	PPO	90	Static-β	-0.000	0.982	0.998	0.507
equity	PPO	120	Sortino	0.000	0.986	0.998	0.511
equity	PPO	120	Momentum-β	0.000	0.986	0.998	0.511
equity	PPO	120	Dynamic-β	0.000	0.986	0.998	0.511
equity	PPO	120	Static-β	0.000	0.986	0.998	0.511
crypto	PPO	30	Sortino	0.001	0.882	0.847	0.422
crypto	PPO	30	Momentum-β	0.001	0.836	0.792	0.403
crypto	PPO	30	Dynamic-β	-0.000	0.990	0.992	0.505
crypto	PPO	30	Static-β	0.000	0.972	0.986	0.494
crypto	PPO	60	Sortino	0.001	0.901	0.851	0.421
crypto	PPO	60	Momentum-β	0.000	0.990	0.992	0.505
crypto	PPO	60	Dynamic-β	0.001	0.902	0.862	0.435
crypto	PPO	60	Static-β	0.001	0.903	0.860	0.431
crypto	PPO	90	Sortino	0.001	0.905	0.858	0.428
crypto	PPO	90	Momentum-β	0.001	0.857	0.808	0.409
crypto	PPO	90	Dynamic-β	0.001	0.895	0.849	0.423
crypto	PPO	90	Static-β	0.000	0.966	0.980	0.482
crypto	PPO	120	Sortino	0.000	0.970	0.984	0.489
crypto	PPO	120	Momentum-β	-0.000	0.989	0.992	0.505
crypto	PPO	120	Dynamic-β	0.001	0.884	0.846	0.422
crypto	PPO	120	Static-β	0.001	0.886	0.852	0.427
macro	PPO	30	Sortino	0.001	0.898	0.854	0.424
macro	PPO	30	Momentum-β	-0.001	0.888	0.845	0.421
macro	PPO	30	Dynamic-β	0.001	0.912	0.862	0.427
macro	PPO	30	Static-β	0.001	0.905	0.856	0.424
macro	PPO	60	Sortino	-0.000	0.980	0.994	0.500
macro	PPO	60	Momentum-β	0.000	0.972	0.988	0.492
macro	PPO	60	Dynamic-β	-0.001	0.887	0.848	0.422
macro	PPO	60	Static-β	-0.000	0.960	0.983	0.488
macro	PPO	90	Sortino	-0.001	0.889	0.845	0.423
macro	PPO	90	Momentum-β	0.000	0.978	0.992	0.497
macro	PPO	90	Dynamic-β	-0.001	0.885	0.842	0.421
macro	PPO	90	Static-β	-0.000	0.964	0.987	0.492
macro	PPO	120	Sortino	-0.000	0.966	0.986	0.491
macro	PPO	120	Momentum-β	0.001	0.906	0.855	0.423
macro	PPO	120	Dynamic-β	-0.001	0.890	0.849	0.424

Continued on next page

Table 1 – Continued from previous page

Domain	Algorithm	Window	Comparator	ΔSharpe	p_t	p_W	p_{boot}
macro	PPO	120	Static-β	-0.001	0.894	0.851	0.425
multi	PPO	30	Sortino	-0.001	0.877	0.838	0.418
multi	PPO	30	Momentum-β	0.001	0.886	0.845	0.420
multi	PPO	30	Dynamic-β	-0.001	0.876	0.836	0.416
multi	PPO	30	Static-β	-0.001	0.868	0.829	0.413
multi	PPO	60	Sortino	0.000	0.977	0.994	0.501
multi	PPO	60	Momentum-β	0.001	0.889	0.848	0.423
multi	PPO	60	Dynamic-β	-0.000	0.979	0.994	0.500
multi	PPO	60	Static-β	-0.001	0.884	0.845	0.422
multi	PPO	90	Sortino	0.001	0.907	0.861	0.429
multi	PPO	90	Momentum-β	0.001	0.896	0.851	0.423
multi	PPO	90	Dynamic-β	-0.001	0.891	0.847	0.422
multi	PPO	90	Static-β	-0.000	0.973	0.991	0.494
multi	PPO	120	Sortino	0.001	0.900	0.852	0.424
multi	PPO	120	Momentum-β	0.001	0.908	0.861	0.430
multi	PPO	120	Dynamic-β	-0.000	0.977	0.993	0.499
multi	PPO	120	Static-β	-0.001	0.886	0.845	0.422
multi	SAC	30	Sortino	0.002	0.822	0.791	0.409
multi	SAC	30	Momentum-β	0.002	0.820	0.786	0.406
multi	SAC	30	Dynamic-β	0.003	0.778	0.745	0.389
multi	SAC	30	Static-β	0.001	0.911	0.867	0.432
multi	SAC	60	Sortino	0.000	0.980	0.995	0.502
multi	SAC	60	Momentum-β	0.000	0.978	0.993	0.499
multi	SAC	60	Dynamic-β	0.002	0.842	0.803	0.411
multi	SAC	60	Static-β	0.000	0.972	0.990	0.494
multi	SAC	90	Sortino	0.001	0.911	0.867	0.435
multi	SAC	90	Momentum-β	0.001	0.921	0.875	0.436
multi	SAC	90	Dynamic-β	0.002	0.853	0.812	0.415
multi	SAC	90	Static-β	0.001	0.917	0.872	0.437
multi	SAC	120	Sortino	-0.001	0.889	0.849	0.424
multi	SAC	120	Momentum-β	0.001	0.934	0.884	0.440
multi	SAC	120	Dynamic-β	0.002	0.853	0.811	0.414
multi	SAC	120	Static-β	0.000	0.977	0.993	0.499
multi	TD3	30	Sortino	0.003	0.767	0.733	0.384
multi	TD3	30	Momentum-β	0.002	0.807	0.773	0.400
multi	TD3	30	Dynamic-β	0.002	0.815	0.781	0.404
multi	TD3	30	Static-β	0.000	0.974	0.992	0.497
multi	TD3	60	Sortino	0.003	0.761	0.728	0.382
multi	TD3	60	Momentum-β	0.002	0.804	0.771	0.397
multi	TD3	60	Dynamic-β	0.001	0.906	0.863	0.433
multi	TD3	60	Static-β	0.001	0.909	0.864	0.433
multi	TD3	90	Sortino	0.003	0.770	0.736	0.386
multi	TD3	90	Momentum-β	0.003	0.759	0.724	0.380
multi	TD3	90	Dynamic-β	-0.000	0.972	0.992	0.498
multi	TD3	90	Static-β	0.001	0.909	0.864	0.432
multi	TD3	120	Sortino	0.003	0.760	0.726	0.381
multi	TD3	120	Momentum-β	0.002	0.806	0.772	0.398
multi	TD3	120	Dynamic-β	0.002	0.813	0.779	0.402
multi	TD3	120	Static-β	0.000	0.982	0.995	0.503
