# Supplementary material for: Factor-based deep reinforcement learning for asset allocation: Comparative analysis of static and dynamic beta reward designs
Source: PLoS One. 2025 Dec 30;20(12):e0332779. doi: 10.1371/journal.pone.0332779 (PMC12753089; doi:10.1371/journal.pone.0332779)
Supplement: S9 Table — (PDF) [file pone.0332779.s009.pdf]

S9 Table. Global BH–FDR significance pattern across assets and algorithms

Asset group	Algorithm	Reward comparator(s)	Global BH–FDR result
Equity	PPO	Sortino, Momentum-β, Dynamic-β, Static-β	Most windows: BH–FDR q-values > 0.05; no reward survives global FDR control.
Equity	SAC / TD3	Sortino, Momentum-β, Dynamic-β, Static-β	Effect sizes are small; all BH–FDR q-values remain above 0.05.
Crypto	PPO	Sortino, Momentum-β, Dynamic-β, Static-β	Several raw p-values < 0.05 (HAC / Wilcoxon), but all corresponding BH–FDR q-values > 0.05; no crypto comparison remains significant after global FDR adjustment.
Crypto	SAC / TD3	Sortino, Momentum-β, Dynamic-β, Static-β	Similar pattern: scattered raw significance, but no reward survives global BH–FDR control.
Macro	PPO	Dynamic-β (core), Static-β (marginal)	Dynamic-β at intermediate windows retains BH–FDR $q < 0.10$ (often < 0.05) in HAC and Wilcoxon tests; Static-β is at best marginal (borderline q-values).
Macro	SAC / TD3	Sortino, Momentum-β, Dynamic-β, Static-β	No consistent global BH–FDR significance; signals remain exploratory rather than confirmatory.
Multi-asset	PPO	Sortino, Momentum-β, Dynamic-β, Static-β	Raw p-values occasionally < 0.05, but global BH–FDR q-values mostly > 0.05; treated as exploratory differences.
Multi-asset	SAC	Sortino, Momentum-β, Dynamic-β	Several window lengths show BH–FDR $q < 0.05$ for Sortino and Momentum-β (sometimes Dynamic-β); these represent the strongest global FDR-adjusted signals.
Multi-asset	TD3	Sortino, Momentum-β (core)	Sortino and Momentum-β maintain BH–FDR $q < 0.05$ for multiple windows; Dynamic-/Static-β are sporadic or non-significant after global FDR correction.
