# Supplementary material for: Factor-based deep reinforcement learning for asset allocation: Comparative analysis of static and dynamic beta reward designs
Source: PLoS One. 2025 Dec 30;20(12):e0332779. doi: 10.1371/journal.pone.0332779 (PMC12753089; doi:10.1371/journal.pone.0332779)
Supplement: S10 Table — (PDF) [file pone.0332779.s010.pdf]

S10 Table. Family-wise BH-FDR adjusted p-values across domains, algorithms, and reward comparators.

Domain	Algorithm	Reward Comparator	BH-FDR p-value	Signif.
crypto	PPO	momentum-β	0.6797	—
crypto	PPO	sortino	0.6797	—
crypto	PPO	dynamic-β	0.6797	—
crypto	PPO	static-β	0.6797	—
macro	PPO	dynamic-β	0.0541	*
macro	PPO	static-β	0.1083	.
multi	SAC	momentum-β	0.0315	**
multi	SAC	dynamic-β	0.0448	**
multi	SAC	sortino	0.0287	**
multi	SAC	static-β	0.0912	*
multi	TD3	sortino	0.0394	**
multi	TD3	momentum-β	0.0475	**
multi	TD3	dynamic-β	0.2117	—
multi	TD3	static-β	0.1881	—

Notes: Significance codes: '***' $p < 0.01$, '**' $p < 0.05$, '*' $p < 0.10$, '.' $p < 0.15$, '—' not significant.
