# Supplementary material for: Factor-based deep reinforcement learning for asset allocation: Comparative analysis of static and dynamic beta reward designs
Source: PLoS One. 2025 Dec 30;20(12):e0332779. doi: 10.1371/journal.pone.0332779 (PMC12753089; doi:10.1371/journal.pone.0332779)
Supplement: S11 Table — (PDF) [file pone.0332779.s011.pdf]

S11 Table. Moving-block bootstrap p-values across alternative block lengths (days).

Domain	Algo	Window	Comparator	Block	MBB p	CI Low	CI High
equity	PPO	60	sortino	10	0.421	-0.000953	0.000983
equity	PPO	60	sortino	20	0.398	-0.000942	0.000881
equity	PPO	60	sortino	40	0.377	-0.000799	0.000755
macro	PPO	90	dynamic-β	10	0.477	-0.000521	0.000495
macro	PPO	90	dynamic-β	20	0.512	-0.000490	0.000428
macro	PPO	90	dynamic-β	40	0.546	-0.000479	0.000419
crypto	PPO	30	sortino	10	0.487	-0.004437	0.004307
crypto	PPO	30	sortino	20	0.445	-0.004169	0.003810
crypto	PPO	30	sortino	40	0.518	-0.003660	0.003450
