# Supplementary material for: Factor-based deep reinforcement learning for asset allocation: Comparative analysis of static and dynamic beta reward designs
Source: PLoS One. 2025 Dec 30;20(12):e0332779. doi: 10.1371/journal.pone.0332779 (PMC12753089; doi:10.1371/journal.pone.0332779)
Supplement: S12 Table — (PDF) [file pone.0332779.s012.pdf]

S12 Table. Subsample robustness of portfolio performance across reward functions.

Domain	Algo	Reward	Sharpe (Early)	Sharpe (Middle)	Sharpe (Late)
equity	PPO	sharpe	0.92	1.10	1.16
equity	PPO	dynamic-β	0.94	1.13	1.18
crypto	PPO	sharpe	0.44	0.35	0.29
crypto	PPO	momentum-β	0.46	0.33	0.31
macro	PPO	sharpe	0.58	0.62	0.66
macro	PPO	dynamic-β	0.61	0.65	0.70
multi	SAC	sortino	1.12	1.25	1.30
multi	TD3	momentum-β	1.08	1.21	1.28

Note: Early = 2015–2018, Middle = 2019–2021, Late = 2022–2025.
